# The Prime Diet Quality Score (PDQS), chronic disease and cause-specific mortality in UK Biobank: a prospective study

**DOI:** 10.1007/s00394-025-03877-6

**Published:** 2026-03-17

**Authors:** Selma Kronsteiner-Gicevic, Alysha S. Thompson, Reynalda Cordova, Martina Gaggl, Jayne V. Woodside, Aedín Cassidy, Sabine Rohrmann, Tilman Kühn

**Affiliations:** 1 Department of Nutritional Sciences, University of Vienna, Vienna, Austria; 2 Center for Public Health, Medical University of Vienna, Vienna, Austria; 3 Institute for Global Food Security & Co-Centre for Sustainable Food Systems, Queen’s University Belfast, Belfast, UK; 4 Centre for Public Health, Queen’s University Belfast, Belfast, UK; 5 Epidemiology, Biostatistics and Prevention Institute, University of Zurich, Zurich, Switzerland; 6 Department of Epidemiology, Medical University of Vienna, Vienna, Austria; 7 University of Applied Sciences, Department for Nutritional, Food and Consumer Studies, Hochschule Fulda, Fulda, Germany

**Keywords:** Diet quality, Cardiovascular disease, Cancer, Type 2 diabetes, Non-alcoholic fatty liver disease, Chronic kidney disease, Chronic obstructive pulmonary disease

## Abstract

**Purpose:**

The Prime Diet Quality Score (PDQS) is a global food-based metric for assessing diet quality. We evaluated PDQS-measured diet quality in relation to mortality and major chronic disease outcomes in the UK Biobank.

**Methods:**

This population-based cohort study included UK adults (40–69 years) recruited in 2006–2010 and followed until 2021. PDQS was derived from ≥ 2 dietary recalls. Multivariable Cox regression estimated hazard ratios (HRs) and 95% confidence intervals (CIs) for total and cause-specific mortality, cardio-metabolic diseases (type 2 diabetes [T2D], cardiovascular disease [CVD], myocardial infarction [MI], stroke), respiratory diseases (chronic obstructive pulmonary disease [COPD], asthma), cancers (total, lung, colorectal, oesophageal, postmenopausal breast, prostate), neurodegenerative diseases (dementia, Parkinson’s, anxiety, depression), non-alcoholic fatty liver disease (NAFLD), chronic kidney disease (CKD), eczema, psoriasis, fracture, and osteoporosis.

**Results:**

Among 124,851 participants (mean age 59 ± 8 years), those in the highest PDQS quartile had lower mortality risks: total (HR 0.80, 95% CI 0.73–0.88), cancer (0.87, 0.77–0.98), respiratory (0.56, 0.37–0.87), and neurodegenerative (0.61, 0.39–0.96). Chronic morbidity risks were lower for T2D (0.73, 0.66–0.81), MI (0.80, 0.71–0.90), ischemic stroke (0.86, 0.74–1.00), COPD (0.80, 0.69–0.94), total cancer (0.94, 0.88–0.99), lung cancer (0.75, 0.58–0.97), anxiety (0.85, 0.78–0.92), NAFLD (0.66, 0.56–0.77), and CKD (0.74, 0.69–0.80).

**Conclusions:**

Higher PDQS scores are linked to reduced mortality and chronic disease risk. PDQS is a practical tool for assessing diet quality in epidemiological research.

**Supplementary Information:**

The online version contains supplementary material available at 10.1007/s00394-025-03877-6.

## Introduction

Diet is a leading modifiable risk factor for premature mortality and morbidity, with greater negative impact than unsafe sex, tobacco, alcohol and illegal drugs’ abuse combined [1, 2]. While low-quality diets have been consistently linked to premature mortality and a range of cardio-metabolic outcomes [3–10], studies of associations of diet quality with neurological and mental disorders, malignant neoplasms, respiratory diseases, and digestive diseases have yielded inconsistent results [11–15]. A number of diet quality indices have been developed to respond to a growing scientific and public health need to describe, measure and guide dietary choice. These indices are based either on scientific synthesis of evidence (e.g., Alternate Healthy Eating Index 2010/AHEI-2010 [16], Healthy Plant-based Diet Index/hPDI [17]), official dietary guidelines (e.g. Healthy Eating Index 2015/HEI-2015 [18]), or specific dietary patterns (e.g., Alternate Mediterranean Diet Score/aMED [19]). Some of these metrics include nutrient-based components (e.g., saturated fatty acids) that might require complex calculations using food composition databases or include specific cultural dietary components (e.g., red wine) that may not be appropriate for all population groups.

The Prime Diet Quality Score (PDQS) [20–27] was developed in response to a global need for an easy-to-use diet quality metric. It allows non-experts to quickly evaluate diet quality and guide healthy dietary choice in the general population, while also considering environmental effects including greenhouse gas production, deforestation, and loss of arable land [28]. It is a fully food-based index that can be easily converted into a stand-alone screener and used for various purposes, ranging from rapid evaluation of patient diets in clinics, to monitoring diet quality on a population level. The PDQS has thus far been evaluated in relation to all-cause mortality, coronary heart disease, gestational diabetes mellitus, gestational hypertension, telomere length, and birthweight, predominantly in US populations [21–23, 25–27]. The UK Biobank study [29, 30] provides a unique opportunity to comprehensively evaluate the PDQS in a prospective cohort of adults in a European country setting. The selection of the health outcomes for evaluation of the PDQS in this study was guided by the 2021 Global Burden of disease data [4]. The top causes of death from noncommunicable diseases (NCDs) and major contributors to disability-adjusted life years (DALYs) [31] were prioritized. This included some outcomes that have been rarely studied in relation to diet quality. The aim of our study was to examine whether a higher diet quality measured by the PDQS is associated with a lower risk of mortality and major chronic morbidity outcomes among UK adults.

## Methods

### Study population

The UK Biobank is a large, population-based prospective cohort of more than 500,000 middle- to older-aged adults (aged 40–69 years at baseline; recruited 2006–2010) [29]. Study participants underwent a detailed assessment at one of the 22 centers throughout the UK. More information about the study protocol has been described elsewhere [30]. The study ethics approval was obtained from the NHS North West-Haydock Research Ethics Committee (ref no. 16/NW/0274). At recruitment, all participants provided electronically-signed written informed consent.

The dataset included data on 502,236 individuals. For the present analysis, we excluded participants who had missing data on diet (*N* = 291,361), implausible energy intakes (*N* = 2,124) [32] or less than two 24-hour diet assessments (*N* = 83,900) [33]. We restricted our analyses to participants with two or more diet assessments as it reduces within-person variability and limits regression dilution in diet-disease relationships, yielding more precise hazard estimates. Characteristics of the participants excluded due to ineligible dietary data are presented in Supplementary Table S1. The analytical dataset therefore contained complete data on 124,851 participants. Depending on the outcome of interest, we also excluded participants with relevant disease at baseline (in-hospital of self-reported interview data) or for whom the outcome occurred between recruitment and the last plausible diet assessment; for mortality analyses, we excluded prevalent CVD, T2D and cancer (Supplementary Fig. S1). The study followed the STROBE guidelines for reporting cohort studies [34].

## Exposure assessment: Prime Diet Quality Score (PDQS)

The PDQS [25–27] is a food-based component diet quality score developed from PrimeScreen [35]. It consists of 23 components, of which 15 “healthy”: dark green leafy vegetables, cruciferous vegetables, deep orange vegetables, other vegetables, deep orange fruits, citrus fruits, other fruits, deep orange tubers, legumes, nuts and seeds, fish, poultry, whole grains, liquid oils and low-fat dairy, seven “unhealthy”: red meats, processed meats, white roots and tubers, refined grains and baked goods, sweets and ice cream, sugar-sweetened beverages (SSBs), and fried foods, and one “neutral” for adults in high-income country settings: eggs. The PDQS, when used as a standalone tool, has been shown to have excellent reliability and relative validity [20, 36]. For this analysis, we constructed the PDQS for each participant using data from the Oxford WebQ tool [37] (Supplementary Table S2). The Oxford WebQ dietary questionnaire [38] was administered up to five times among the UK Biobank study participants between 2009 and 2012. The questionnaire asked about the frequency of consumption of up to 206 foods and 32 drinks over the past 24 h [39]. The Oxford WebQ has been validated previously to represent approximate and true dietary intake. In this study, participants who completed two or more ‘eligible’ diet recalls were included [40, 41]. A diet assessment was considered valid if the reported total energy intake ranged between 2092 kJ and 14,644 kJ for women and between 3347 kJ and 17,573 kJ for men. The details of the scoring procedure are included in the Supplementary Methods 1. To calculate the total PDQS value for each participant, each component was assigned 0, 1 or 2 points based on the total estimated consumed amount of each component [25]. The possible PDQS range is 2–46.

## Outcome ascertainment

The outcome ascertainment in the UK Biobank is described in Supplementary Methods 2. In short, mortality status was ascertained by linkage to the death registries [30], cancer diagnoses through record linkage to national cancer registries in England [42], Wales and Scotland, while hospital admission and self-reported data were used for other morbidities.

## Covariate assessment

Data on age, sex, ethnicity, Townsend deprivation index, education, tobacco and alcohol use, physical activity, and medications’ use were obtained from the baseline questionnaire. The body mass index (BMI) was ascertained by trained staff during anthropometric measurements at baseline [30]. Disease status at baseline was obtained both from baseline interview and hospital data. Finally, total energy intake was calculated from the Oxford WebQ [37]. Further details on covariates are given in the Supplementary Table S3.

### Statistical analysis

For the analysis, the PDQS was first categorized into quartiles (with Q1 as the reference quartile and the Q4 as the highest diet quality quartile) and standardised to 1-standard deviation(SD) increase by dividing the continuous PDQS value by its SD. Baseline characteristics of the study sample participants were expressed as frequencies for categorical and mean(SD) values for continuous variables, both as totals and across the quartile of the PDQS. We used Cox proportional hazards models, with age (age-at-recruitment to age-at-event) as the time variable, to evaluate associations of the PDQS quartiles with mortality and morbidity outcomes. Models were first adjusted for sex and ethnicity, and then for a number of other demographic, lifestyle and health variables based on subject knowledge (*Supplementary Methods 3*). Dummy indicator variables were created for missing values on covariates to preserve the sample size (*Supplementary Methods 4*).

As a secondary analysis, we evaluated effect modification by sex, age, education (low vs. other), BMI (< 25 vs. ≥25 kg/m^2^), Townsend deprivation index (low vs. high), smoking status (never vs. ever) and the polygenic risk scores (PRS) (low, medium, high) by adding the interaction term to fully adjusted models. We used the data on polygenic risk scores available from the UK Biobank at the time of analysis, specifically for Alzheimer´s and Parkinson’s diseases, asthma, colorectal, breast and prostate cancers, CVD, ischemic stroke, and T2D. The interaction terms were created by multiplying the PDQS 1-SD increase by the dichotomized/trichotomized covariates of interest. Only statistically significant p-interactions were reported. We conducted several sensitivity analyses: to evaluate potential reverse causation, we ran all main analyses after excluding the first two years of follow-up; to further adjust for any residual effect of smoking, we excluded current smokers from the COPD models, and evaluated heterogeneity of associations between PDQS and COPD among current, past and never smokers. Cubic splines with knots at quintile medians were used to evaluate potential non-linearity in associations between the PDQS and main health outcomes. To test the reproducibility of the PDQS over time, we calculated intraclass coefficients (ICCs) by comparing the average values from the second and third diet assessments with those from the fourth and fifth, following a previously applied approach [43]. The ICCs were obtained from a mixed-effects model (%ICC9 macro) [44].

The analyses were performed in SAS 9.4 for Windows (SAS Institute Inc.) and Stata 17.0 (StataCorp LLC). All tests were two-sided with α < 0.05. The linear-test-for-trend and interaction test P-values were corrected for multiple comparisons using the Benjamini-Hochberg procedure [45] for controlling for the false discovery rate at 5% level across mortality and morbidity outcomes.

## Results

### Study sample characteristics

The study sample included 124,851 participants who completed at least two eligible dietary recalls (mean[SD] number of completed recalls: 2.9[0.9]) (Supplementary Fig. S1). At baseline, the sample comprised 44% male and 91% white participants, with a mean(SD) age of 59(8) years. Additionally, 52% of participants had a higher education degree (Table 1). The PDQS score was normally distributed across the participants, with a mean(SD) of 20(4) of possible 2–46 points (Supplementary Fig. S2).

**Table 1:** Baseline characteristics of the UK Biobank study sample participants (*N* = 124,851) across quartiles of the Prime Diet Quality Score (PDQS)

Characteristic	Total	PDQS quartile			
		Q1	Q2	Q3	Q4
n	124,851	39,955	24,253	33,158	27,485
PDQS score, mean (SD)	20 (4)	16 (2)	20 (0.5)	22 (1)	26 (2)
Sex, n (%)					
Male	55,151 (44)	21,898 (55)	11,118 (46)	13,410 (40)	8,725 (32)
Female	69,700 (56)	18,057 (45)	13,135 (54)	19,748 (60)	18,760 (68)
Age at recruitment, years, mean (SD)	59 (8)	58 (8)	59 (8)	59 (8)	60 (7)
*Ethnicity, n (%)*					
White	114,057 (91)	37,037 (93)	22,245 (92)	30,128 (91)	24,647 (90)
Mixed	3,449 (3)	1,120 (3)	687 (3)	880 (3)	762 (3)
Asian	5,752 (5)	1,366 (4)	1,057 (4)	1,692 (5)	1,637 (5)
Black	454 (0.5)	120 (0.5)	84 (0.4)	125 (0.4)	125 (0.4)
Other	723 (0.5)	178 (0.5)	115 (0.6)	216 (0.6)	214 (0.6)
Townsend deprivation index, mean (SD)	−1.6 (3)	−1.6 (3)	−1.7 (3)	−1.7 (3)	−1.6 (3)
*Education level, n (%)*					
Low	31,990 (26)^a^	10,969 (27)	6,367 (26)	8,263 (25)	6,391 (23)
Medium	19,972 (16)	7,429 (19)	3,921 (16)	4,827 (15)	3,795 (14)
High	64,396 (52)	18,113 (45)	12,376 (51)	17,990 (54)	15,917 (58)
*Smoking status, n (%)*					
Never	71,340 (57)^a^	22,164 (55)	13,856 (57)	19,241 (58)	16,079 (59)
Previous	44,660 (36)	13,792 (35)	8,750 (36)	11,965 (36)	10,153 (37)
Current	8,584 (7)	3,906 (10)	1,596 (7)	1,883 (6)	1,199 (4)
Alcohol use, g/day, mean (SD)	17 (20)	19 (22)	18 (20)	17 (19)	15 (17)
Physical activity, MET hr/wk, mean (SD)	31 (38)	29 (39)	30 (38)	31 (37)	35 (39)
Total energy intake, kJ/d	8,545 (1,959)	8,759 (2,041)	8,517 (1,952)	8,425 (1,907)	8,403 (1,877)
*Medication use, n (%)*					
Blood thinners	16,442 (13)	5,590 (14)	3,237 (13)	4,248 (13)	3,367 (12)
NSAID including aspirin	32,580 (26)	11,004 (28)	3,562 (26)	8,470 (26)	6,742 (25)
BMI, mean (SD)	27 (5)	27 (5)	27 (5)	26 (4)	26 (4)
Cancer at baseline^b^, n (%)	10,119 (8)	2,914 (7)	1,863 (8)	2,806 (9)	2,536 (9)
CVD at baseline^b^, n (%)	4,676 (4)	1,705 (4)	920 (4)	1,167 (4)	884 (3)
T2D at baseline^b^, n (%)	4,839 (4)	1,846 (5)	927 (4)	1,199 (4)	867 (3)

^a^ Counts by category may not sum to the total due to missing data for some covariates

^b^ Either hospital in-patient or self-reported data from verbal interviews at baseline

At recruitment, participants in the highest PDQS quartile (Q4) were older, more physically active, consumed less alcohol, had lower total energy intakes and a lower BMI, were more likely to be female, non-white, highly educated and non-smokers compared to participants in the lowest quartile (Q1). Participants in the highest PDQS quartile were also less likely to use blood thinners or nonsteroidal anti-inflammatory drugs (NSAIDs), had a lower prevalence of CVDs and T2D, though a higher prevalence of cancer at baseline compared to the lowest quartile (Q1). The reproducibility of the PDQS was very good, with an ICC of 0.78 (95% CI: 0.77, 0.78) (Supplementary Table S4).

## PDQS and mortality

**Fig. 1:**
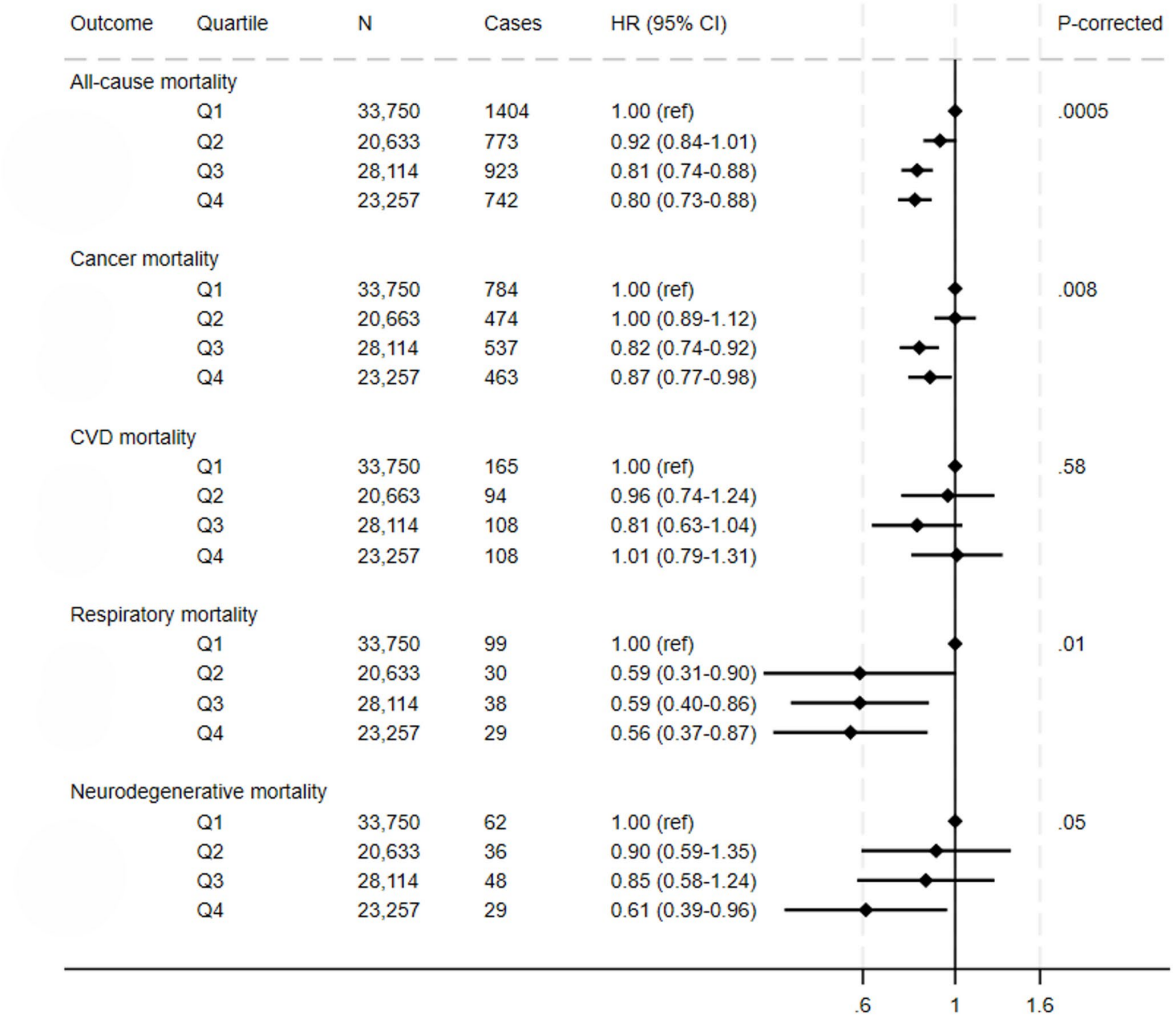
Multivariable adjusted hazard ratios and 95% CIs for mortality (all-cause, cancer, cardiovascular, respiratory and neurodegenerative) (*N* = 105,754) across Prime Diet Quality Score (PDQS) quartiles. All models used age as the underlying time variable and were adjusted for sex, ethnicity, social deprivation index, education level, smoking status, physical activity, alcohol consumption, use of blood thinning medications, use of NSAIDs, total energy intake and BMI, and stratified by region; total mortality models were further adjusted for CVD and cancer at baseline, CVD mortality model for CVD and T2D at baseline, other models for cancer at baseline; respiratory mortality model was also adjusted for smoking intensity and pack years of smoking

In fully-adjusted models, participants with highest PDQS values (Q4) had a 20% lower risk of all-cause mortality compared to the participants with the lowest PDQS values (Q1) (HR, 0.80 [95%CI: 0.73, 0.88]; corrected P-trend = 0.0005) (Fig. 1, Supplementary Table S5). These participants also had a 13% lower cancer mortality (HR, 0.87 [95%CI: 0.77, 0.98]; corrected P-trend = 0.008), a 44% lower respiratory mortality risk (HR, 0.56 [95%CI: 0.37, 0.87]; corrected P-trend = 0.01), and a 39% lower neurodegenerative mortality risk (HR, 0.61 [95%CI: 0.39, 0.96] compared to the participants in the lowest PDQS quartile. The association between PDQS and CVD mortality was null.

### PDQS and cardiometabolic disease

**Fig. 2:**
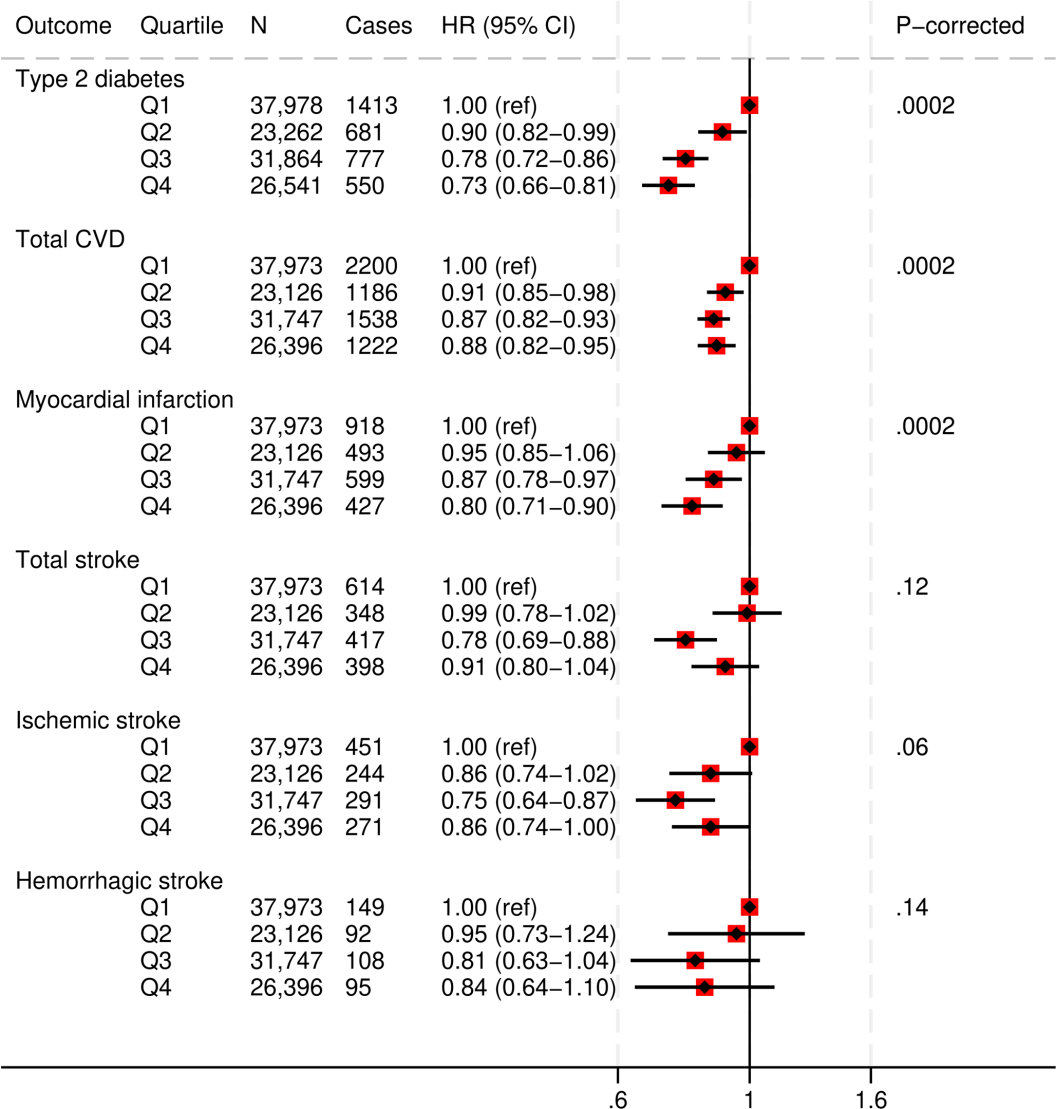
Multivariable adjusted hazard ratios and 95% CIs for cardiometabolic disease outcomes across Prime Diet Quality Score (PDQS) quartiles.All models used age as the underlying time variable and were adjusted for sex, ethnicity, social deprivation index, education level, smoking status, physical activity, alcohol consumption, use of blood thinning medications, use of NSAIDs, total energy intake and BMI, and stratified by region; CVD models were further adjusted for T2D and cancer at baseline, and T2D for CVD and cancer at baseline

Higher PDQS scores (Q4) were associated with a 27% lower diabetes (HR, 0.73 [95%CI: 0.66, 0.81]; corrected P-trend = 0.0002), 12% lower any CVD (HR, 0.88 [95%CI: 0.82, 0.95]; corrected P-trend = 0.0002), 20% lower myocardial infarction (HR, 0.80 [95%CI: 0.71, 0.90]; corrected P-trend = 0.0002), and 14% lower ischemic stroke risk (HR, 0.86 [95%CI: 0.74, 1.00]; corrected P-trend = 0.06), compared to the lowest quartile (Q1) PDQS scores (Fig. 2 and Supplementary Table S6). The associations with total and hemorrhagic stroke were not statistically significant.

### PDQS and respiratory disease

**Fig. 3:**
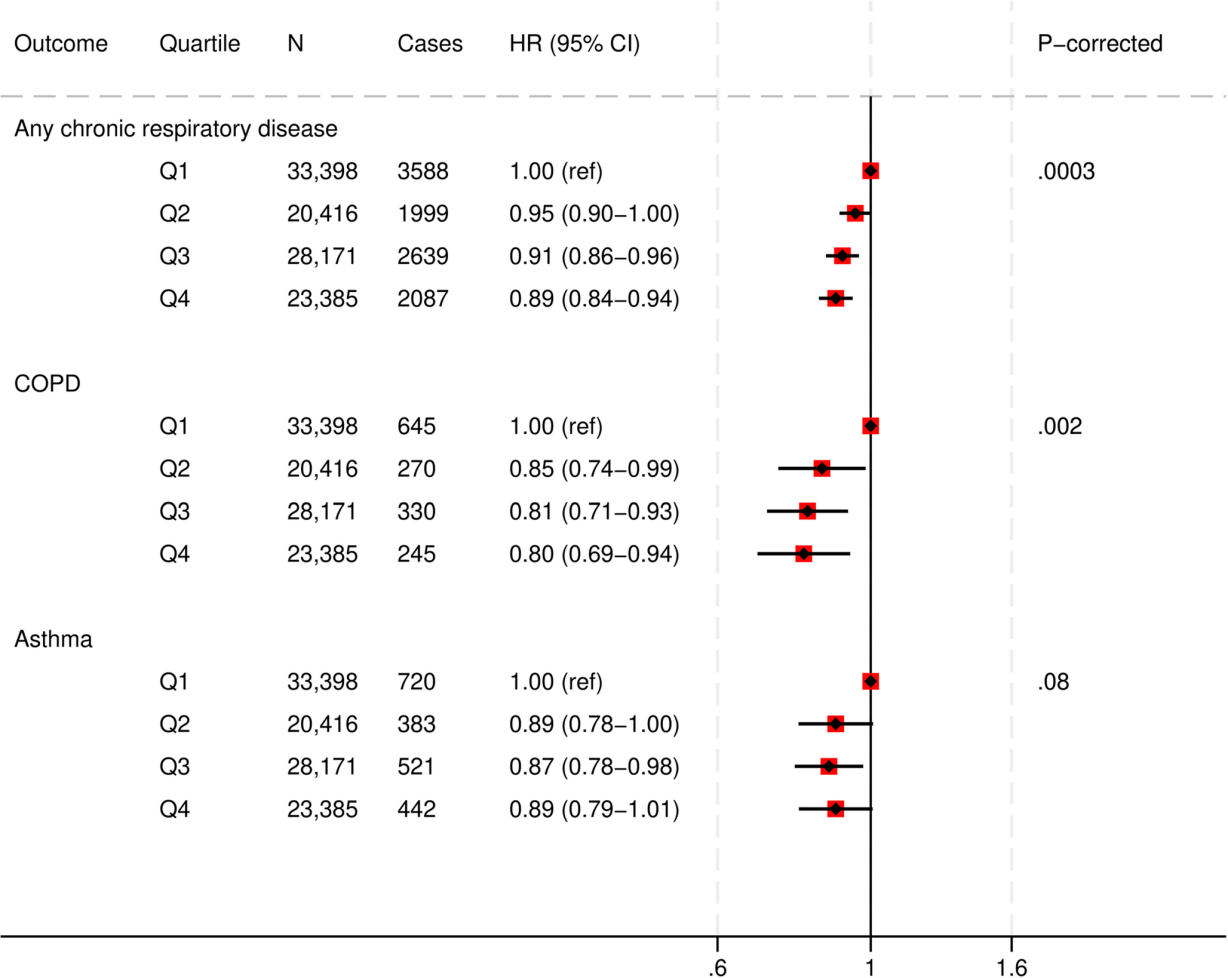
Multivariable adjusted hazard ratios and 95% CIs for chronic respiratory disease outcomes across Prime Diet Quality Score (PDQS) quartiles.All models used age as the underlying time variable and were adjusted for sex, ethnicity, social deprivation index, education level, smoking status, smoking intensity, pack years of smoking, physical activity, alcohol consumption, use of blood thinning medications, use of NSAIDs, total energy intake, BMI, cancer, CVD and type 2 diabetes at baseline, and stratified by region.

Greater PDQS adherence (Q4 vs. Q1) was associated with a 11% lower risk of any chronic respiratory disease (HR, 0.89 [95%CI: 0.84, 0.94]; corrected P-trend = 0.0003), and a 20% lower risk of COPD (HR, 0.80 [95%CI: 0.69, 0.94]; corrected P-trend = 0.002) in fully adjusted models. No association between the PDQS values and asthma was found in fully adjusted models. (Fig. 3 and Supplementary Table S7). The COPD association did not considerably change when current smokers were excluded (HR, 0.82 [95%CI: 0.69, 0.97]; corrected P-trend = 0.01) (Supplementary Table S8).

### PDQS and cancer

**Fig. 4:**
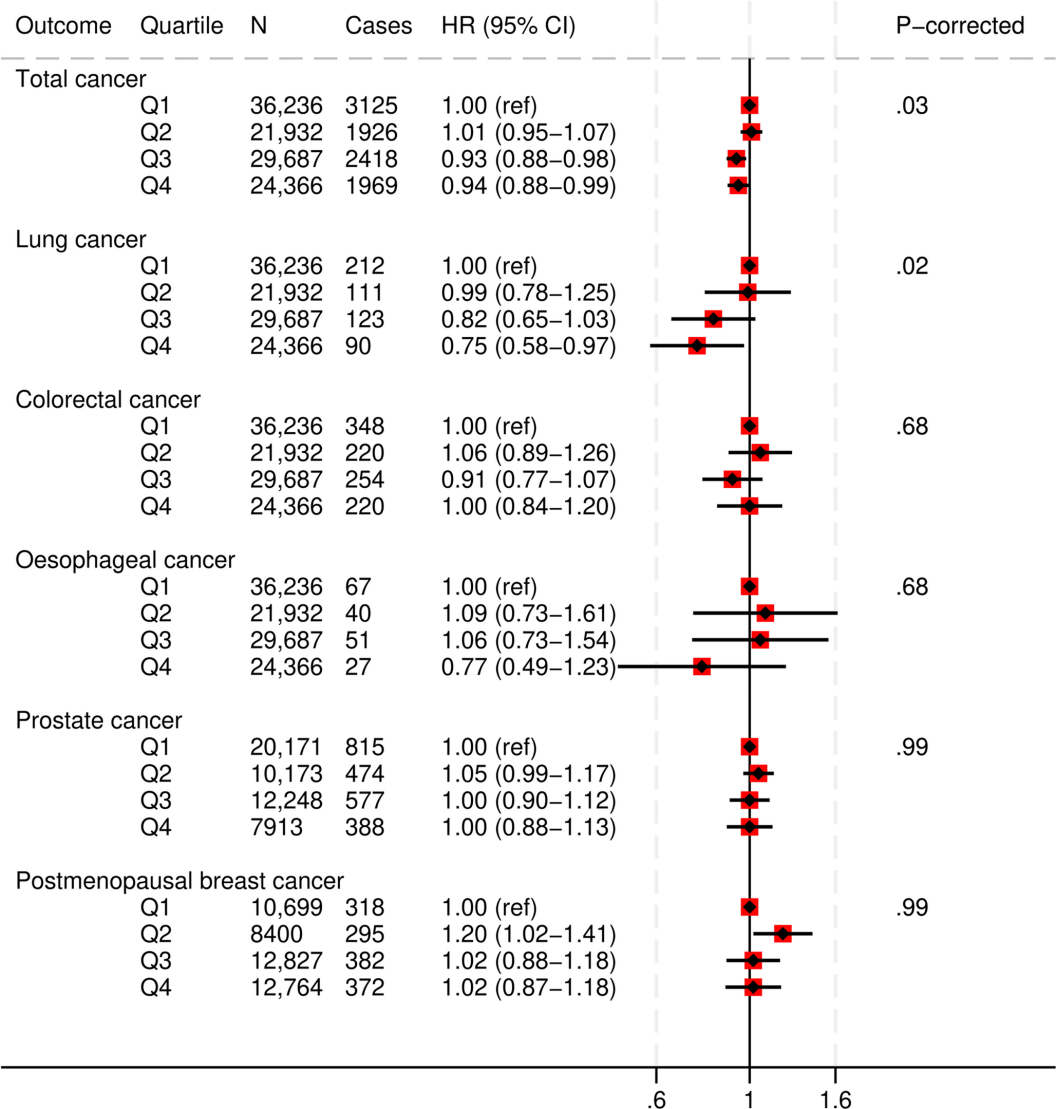
Multivariable adjusted hazard ratios and 95% CIs for cancer outcomes across Prime Diet Quality Score (PDQS) quartiles. All models used age as the underlying time variable and were adjusted for sex, ethnicity, social deprivation index, education level, smoking status, physical activity, alcohol consumption, use of blood thinning medications, use of NSAIDs, use of HRT and menopausal status (any cancer, women only), total energy intake, BMI, CVD and type 2 diabetes at baseline, and stratified by region. Lung cancer model was also adjusted for smoking intensity and pack years of smoking

In fully-adjusted models, a higher PDQS score was associated with a 6% lower risk of cancer (total) (HR, 0.94 [95%CI: 0.88, 0.99]; corrected P-trend = 0.03), and a 25% lower risk of lung cancer (HR, 0.75 [95%CI: 0.58, 0.97]; corrected P-trend = 0.02) (Fig. 4 and Supplementary Table S9). We found no evidence of an association between the highest PDQS values and the risk of colorectal, oesophageal, prostate, or postmenopausal breast cancer.

### PDQS and neurological and psychological disease

**Fig. 5:**
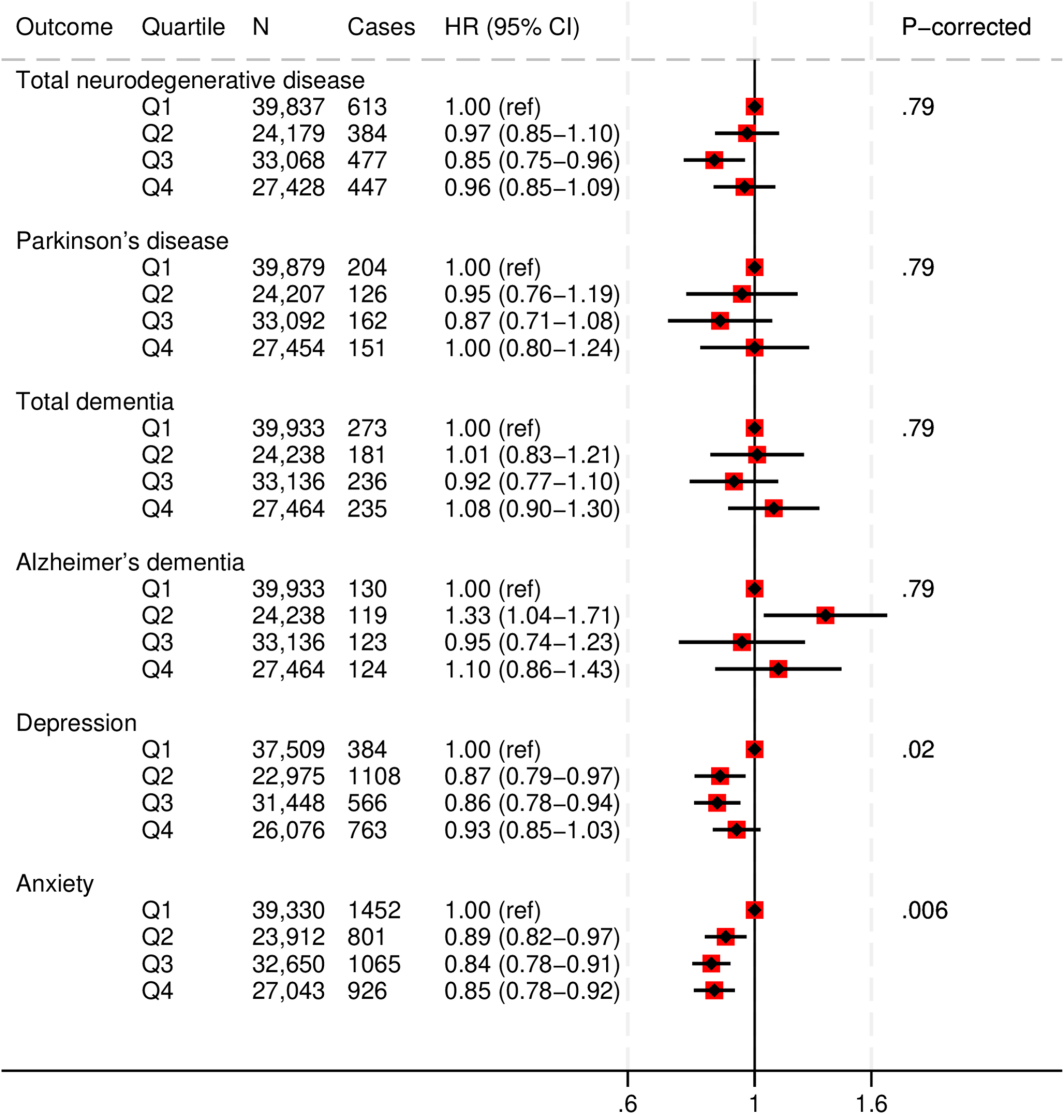
Multivariable adjusted hazard ratios and 95% CIs for neurodegenerative disease outcomesacross Prime Diet Quality Score (PDQS) quartiles. All models used age as the underlying time variable and were adjusted for sex, ethnicity, social deprivation index, education level, smoking status, physical activity, alcohol consumption, use of blood thinning medications, use of nonsteroidal anti-inflammatory drugs, TEI, body mass index, CVD, cancer or T2D at baseline, and stratified by region.

Our analyses did not find associations between the PDQS and neurodegenerative disease outcomes or depression. In fully adjusted models, a higher PDQS value was associated with a 15% lower risk of anxiety (HR, 0.85 [95%CI: 0.78, 0.92]; corrected P-trend = 0.006) (Fig. 5 and Supplementary Table S10).

### PDQS, non-alcoholic fatty liver disease (NAFLD) and chronic kidney disease (CKD)

**Fig. 6:**
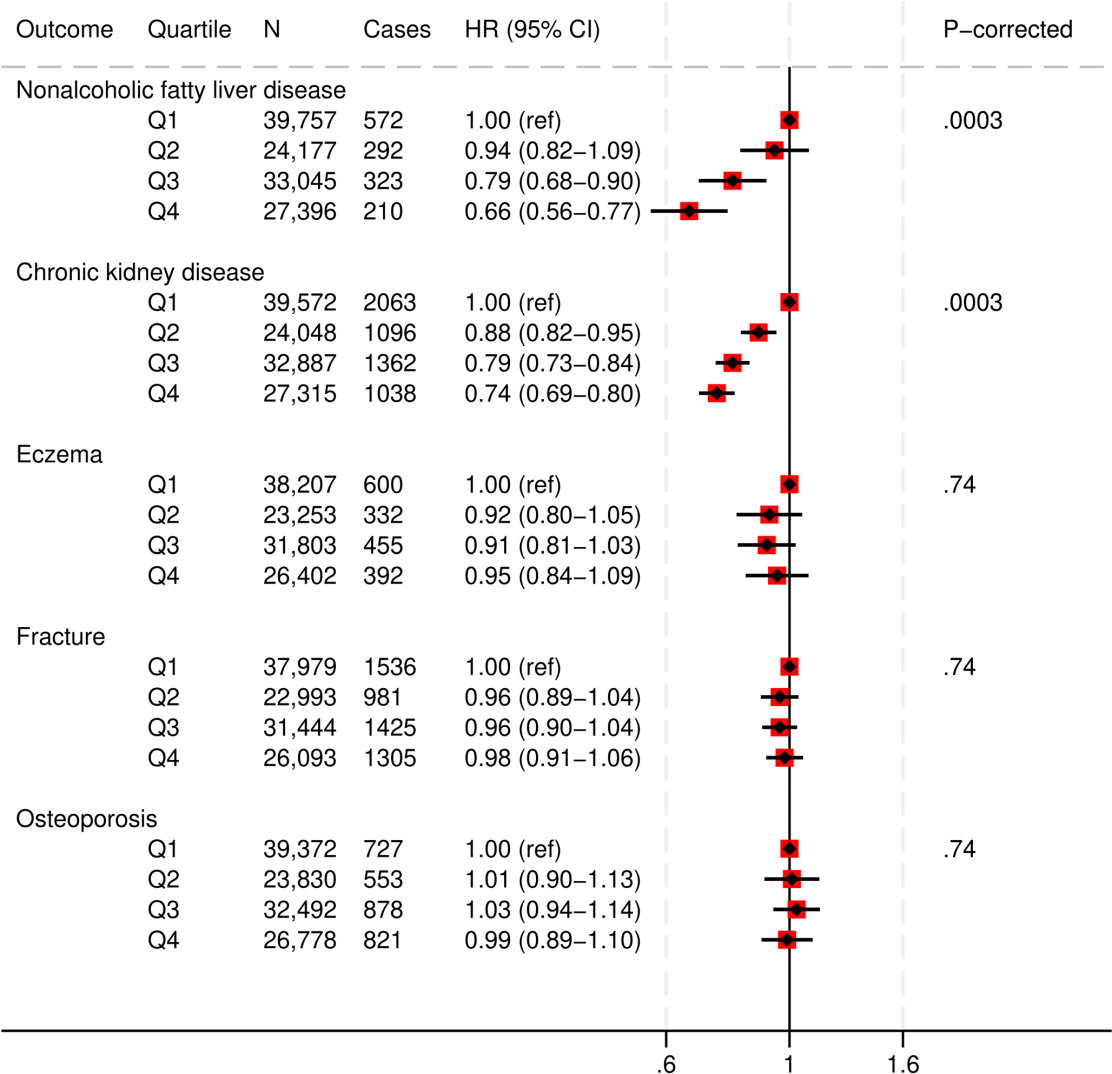
Multivariable adjusted hazard ratios and 95% CIs for other chronic disease outcomes across Prime Diet Quality Score (PDQS) quartiles. All models used age as the underlying time variable and were adjusted for sex, ethnicity, social deprivation index, education level, smoking status, physical activity, alcohol consumption, use of blood thinning medications, use of NSAIDs, total energy intake, BMI, CVD, cancer or type 2 diabetes at baseline, and stratified by region; fracture and osteoporosis models were also adjusted for multivitamin and multimineral use

Participants in the top quartile (Q4) of the PDQS had a 34% lower risk of NAFLD (HR, 0.66 [95%CI: 0.56, 0.77]; corrected P-trend = 0.0003) and a 26% lower risk of CKD (HR, 0.74 [95%CI: 0.69, 0.80]; corrected P-trend = 0.0003) compared to the lowest quartile (Q1) (Fig. 6 and Supplementary Table S11).

### PDQS, fracture risk, osteoporosis and skin conditions

We found no evidence of associations between the PDQS and osteoporosis, fracture risk or eczema/psoriasis (Fig. 6 and Supplementary Table S11).

### Ancillary analyses

The majority of subgroup analyses did not suggest differential associations across strata of covariates, with the exception of several associations where we observed statistically significant interactions between the PDQS and covariates (Supplementary Table S12). PDQS associations with mortality risk were somewhat stronger in females, individuals living in areas with a high Townsend deprivation index, low education, and current/past smokers. Also, associations with the CKD appeared stronger among older individuals and persons living in areas with a high deprivation index. None of the associations between the PDQS and the main health outcomes was significantly non-linear in fully adjusted models with restricted cubic splines (Supplementary Fig S3). The results remained similar after excluding the first two years of follow-up (Supplementary Table S13). Finally, the test for heterogeneity in the association between PDQS and COPD based on smoking status was not significant (Supplementary Table S8).

## Discussion

In this large prospective cohort study, we found that a higher PDQS was associated with a lower risk of total, respiratory, neurodegenerative, and cancer mortality, and a lower risk of major chronic diseases, including total cardiovascular disease, myocardial infarction, ischemic stroke, type 2 diabetes, total chronic respiratory disease, COPD, total cancer, lung cancer, non-alcoholic fatty liver disease, chronic kidney disease, and anxiety. However, the PDQS was not associated with CVD–related mortality, total and hemorrhagic stroke, colorectal, prostate or breast cancer, asthma, Parkinson’s disease, Alzheimer’s or total dementia, depression, eczema/psoriasis, osteoporosis or fracture risk.

The PDQS showed robust associations across the majority of outcomes by sex, age group, BMI, education, deprivation score, smoking status or polygenic risk score. Stronger associations with mortality among women, older individuals, low education, high deprivation index, and past/present smokers suggest that vulnerable population groups may especially benefit from a higher diet quality. Nevertheless, healthy diet remains important for everyone, regardless of their baseline risk. Our findings are in line with the literature on other diet quality scores and all-cause mortality [3, 5], including the PDQS mortality analysis among US adults in NHANES [25] and analyses in the UK Biobank [46, 47]. Similarly, the results for type 2 diabetes and cardiovascular disease findings correspond with the PDQS evaluations in US [26, 27, 48] and European [49] cohorts, and with evaluations of other diet quality scores in the UK Biobank [13, 50, 51] and US cohorts [10]. Our findings also support the literature on diet quality, as operationalized by other diet quality scores, and total cancer risk [13, 52], lung cancer [53, 54], COPD [13, 55], CKD [13, 56] and NAFLD [13, 57, 58] in both UK Biobank and other cohorts.

While results for total stroke were not statistically significant, we found an inverse association with ischemic stroke and a null association with hemorrhagic stroke, consistent with an expanding body of literature [59, 60]. For specific tumor sites, our findings correspond with the prostate cancer findings from the World Cancer Research Fund/American Institute for Cancer Research (WCRF/AICR) score assessment in the UK Biobank [61] which found no association between adherence to the 2018 WCRF/AICR recommendations [62] and incident prostate cancer, but not for colorectal and breast cancer, where Malcomson et al. observed inverse associations with higher WCRF/AICR adherence, whereas our PDQS-based analyses were null. These differences may reflect that the WCRF/AICR score operationalizes a joint lifestyle construct (diet quality together with body weight, physical activity, and alcohol, and breastfeeding where applicable), while our PDQS captures diet quality alone using a brief screener. Our null findings for Parkinson’s disease are in contrast with a recent hPDI analysis in the UK Biobank [63]; one possible explanation for this finding could be that inverse associations are driven by specific dietary component such as tea or coffee [11] not included in the PDQS. While inverse associations between diet quality and depression were found in some, but not all studies [64], reverse causation cannot be ruled out in these studies as persons with depressive symptoms and anxiety might change their diet long before the official diagnosis; our null findings correspond with the PDQS analysis of depression in the prospective SUN cohort [21]. For dementia, whereas some diet scores were associated with less signs of cognitive decline [65, 66], these associations may be attributed specifically to the use of olive oil and not to the overall diet quality [67]. Finally, our null findings for osteoporosis and fracture risk are in line with data from other diet indices in the UK Biobank [13].

As a fully food-based and easy-to-calculate score, PDQS can be easily converted to a stand-alone diet screener and applied in both clinical and public health settings and is not specific for certain populations or dietary patterns. Notably, the ICC of 0.78 for the PDQS in our study based on two 24-hour dietary assessments is similarly high as for best-performing diet quality indices based on detailed FFQs [68]. Moreover, its reliability was superior to that of common, but more specific diet quality indices used in the UK Biobank [43, 50]. This underlines its usefulness in the screening contexts where detailed dietary assessments may not be feasible.

### Strengths and limitations

To our knowledge, this is the first comprehensive study of the PDQS in relation to mortality and large number of morbidity outcomes, including some of those rarely studied in relation to diet quality, in a large population-based prospective cohort. Among the strengths of our study are a large study sample, sufficient case numbers for most outcomes and an extensive number of covariates.

The results of our study should be considered in light of the following limitations. First, given the observational nature of our study we cannot rule out residual confounding or make causal conclusions. Second, UK Biobank has a low response rate to recruitment invitation (about 5%), with participants being predominantly Caucasian, older, less likely to smoke, drink alcohol daily and be obese compared to the general UK population [69]. Additionally, we excluded participants with certain disease diagnoses at baseline and those who did not complete at least two diet assessments. These exclusions, along with other study limitations, restrict the generalizability of our findings to both the general UK population and non-UK populations. Future studies should evaluate the PDQS in non-Caucasian populations, and individuals with chronic diseases. Third, the inpatient and mortality data in UK Biobank do not differentiate between aggressive and non-aggressive prostate cancer, limiting our ability to detect any association of diet quality with this outcome. Fourth, in some cases, such as for dementia, presence of preclinical disease developing a decade or more before diagnosis, might have limited participant capability to correctly report diet intake; while these results remained unchanged after excluding the first two years of follow-up, they should still be interpreted with caution.

## Conclusions

The results of this study suggest that a high-quality diet that promotes consumption of a variety of fruits and vegetables, nuts, seeds, legumes, whole grains and fish, and discourages consumption of red meat, refined grains, and sweetened beverages and foods was associated with a lower risk of mortality and a number of chronic disease outcomes among UK adults. This study demonstrates that PDQS effectively captures most diet-health outcome associations that are identified by other, more complex, diet indices in the UK Biobank. As such, it can serve as a feasible and user-friendly diet quality metric for applications in epidemiological research and public health surveillance.

## Supplementary Information

Below is the link to the electronic supplementary material.

Supplementary Material 1

## Abbreviations

BMI: Body mass index
MET: Metabolic equivalent task
NSAID: Nonsteroidal anti-inflammatory drugs
CVD: Cardiovascular disease
T2D: Type 2 diabetes
