# Supplementary material for: The Prime Diet Quality Score (PDQS), chronic disease and cause-specific mortality in UK Biobank: a prospective study
Source: Eur J Nutr. 2026 Mar 17;65(3):99. doi: 10.1007/s00394-025-03877-6 (PMC12995929; doi:10.1007/s00394-025-03877-6)
Supplement: Supplementary file 1 — Supplementary Material 1 [file 394_2025_3877_MOESM1_ESM.docx]

*Kronsteiner-Gicevic et al. The Prime Diet Quality Score (PDQS), Chronic Disease and Cause-Specific Mortality in UK Biobank: A prospective study*

**Supplementary Web Content**

**Supplementary Methods 1** Prime Diet Quality Score (PDQS) components and scoring method

**Supplementary Methods 2** Outcome ascertainment

**Supplementary Methods 3** Multivariable adjustment

**Supplementary Methods** **4** Missing data on covariates

**Supplementary Table S1** Comparison of baseline characteristics of the UK Biobank excluded participants (N=377,385) with included participants (N=124,851)

**Supplementary Table S2** Prime Diet Quality Score (PDQS) components and UK Biobank food items included

**Supplementary Table S3** Covariate coding and categorisation information

**Supplementary Table S4** Intraclass correlation coefficients (ICCs) for reproducibility of the PDQS over time

**Supplementary Table S5** Hazard ratios and 95% confidence intervals between the Prime Diet Quality Score (PDQS) and mortality in UK Biobank

**Supplementary Table S6** Hazard ratios and 95% confidence intervals between the Prime Diet Quality Score (PDQS) and cardiometabolic disease outcomes in UK Biobank

**Supplementary Table S7** Hazard ratios and 95% confidence intervals between the Prime Diet Quality Score (PDQS) and chronic respiratory outcomes in UK Biobank

**Supplementary Table S8** Hazard ratios and 95% confidence intervals between the Prime Diet Quality Score (PDQS) and COPD across smoking status categories in UK Biobank

**Supplementary Table S9** Hazard ratios and 95% confidence intervals between the Prime Diet Quality Score (PDQS) and cancer in UK Biobank

**Supplementary Table S10** Hazard ratios and 95% confidence intervals between the Prime Diet Quality Score (PDQS) and neurological and psychological disorders in UK Biobank

**Supplementary Table S11** Hazard ratios and 95% confidence intervals between the Prime Diet Quality Score (PDQS) and other chronic diseases in UK Biobank

**Supplementary Table S12** Hazard ratios and 95% confidence intervals between the top quartile of the Prime Diet Quality Score (PDQS) and mortality and morbidity outcomes among UK Biobank population subgroups

**Supplementary Table S13** Sensitivity analysis showing hazard ratios and 95% confidence intervals between the Prime Diet Quality Score (PDQS) and health outcomes in UK Biobank after removing the first 2 years of follow-up

**Supplementary Fig. S1** Flow diagram of UK Biobank participants included in analysis

**Supplementary Fig. S2** Distribution of the total Prime Diet Quality Score (PDQS)

**Supplementary Fig. S3** Cubic spline graph of fully adjusted associations between the Prime Diet Quality Score (PDQS) with selected health outcomes.

**Supplementary references**

*This supplementary material has been provided by the authors to give readers additional information about their work.*

**Supplementary Web Content**

**Supplementary Methods 1** Prime Diet Quality Score (PDQS) components and scoring method

The PDQS consists of 23 components, of which 15 “healthy”: dark green leafy vegetables, cruciferous vegetables, deep orange vegetables, other vegetables, deep orange fruits, citrus fruits, other fruits, deep orange tubers, legumes, nuts and seeds, fish, poultry, whole grains, liquid oils and low-fat dairy, seven “unhealthy”: red meats, processed meats, white roots and tubers, refined grains and baked goods, sweets and ice cream, sugar-sweetened beverages (SSBs), and fried foods, and one “neutral” for adults in high-income country settings: eggs. The PDQS, when used as a standalone tool, has been shown to have excellent reliability and relative validity [1, 2].

Data on consumed individual food frequencies were converted to amounts in g/ml by multiplying frequencies by standard portion sizes and summarized to obtain total amounts within each PDQS component in grams/ml per day. The PDQS component values were then averaged over two or more days. The PDQS cut-offs [3] were used to convert the averaged “amount per day” values into “0”, “1” or “2” points for healthy, and “2”, “1” or “0” for unhealthy components based on the cut-offs used in our previous studies [3]. Data on vegetable oils were not available in portions per day in UK Biobank, hence we could not derive full scores for this component. Instead, participants were assigned two points if they reported using any liquid oil (e.g. olive oil) and “0” points if they reported consuming no oils. Finally, eggs were treated as a neutral component and all participants were assigned a score of “2” in line with the PDQS scoring guidelines[3]. PDQS was computed from a short-form screener using food-group frequencies; total energy intake is neither estimated by this instrument nor used in score construction, and the PDQS scoring is not sex-specific; sex was included as a covariate in all models. The possible PDQS range is 2-46.

**Supplementary Methods 2** **Outcome ascertainment**

The outcomes in the current study were total and cause-specific mortality (cancer, CVD, respiratory and neurodegenerative mortality) and incidence of cardio-metabolic diseases including type 2 diabetes (T2D), cardiovascular disease (CVD), myocardial infarction, ischemic and hemorrhagic stroke, respiratory diseases (total chronic, chronic obstructive pulmonary disease/COPD, asthma), cancers (total, lung, colorectal, oesophageal, postmenopausal breast and prostate), neurological and mental disorders (total, Parkinson’s, dementia including Alzheimer’s, depression and anxiety), non-alcoholic fatty liver disease (NAFLD), chronic kidney disease (CKD), eczema, psoriasis, fracture and osteoporosis. Date of death was obtained from death certificates held by the National Health Service (NHS) Information Centre (England and Wales) and the NHS Central Register Scotland (Scotland). Death data was available until 30^th^ November 2022 for England, Scotland, and Wales. Cancer diagnoses and dates were ascertained through the National Cancer Registries (England, Scotland, and Wales). Cancer data were available until 31^st^ December 2016 for Wales, 31^st^ December 2020 for England, and 30^th^ November 2021 for Scotland. Dates and causes of hospital admissions for other morbidities were identified through record linkage to Health Episode Statistics (England), the Patient Episode Database (Wales) and the Scottish Morbidity Records (Scotland). Hospital admissions follow-up data for other morbidities were available until 31^st^ October 2022 for England, 31^st^ August 2022 for Scotland, and 31^st^ May 2022 for Wales. Incident outcomes were defined as a hospital admission or death identified through primary or secondary diagnosis codes using International Classification of Diseases, Tenth Revision (ICD-10), or equivalent ninth revision (ICD-9) codes, as follows: type 2 diabetes (E11), CVD (I20-I23, I24.1, I25.1, I60, I63, I64), myocardial infarction (I21-23, I24.1, I25.1), ischemic stroke (I63), hemorrhagic stroke (I61), chronic respiratory diseases (total (J30-J98), chronic obstructive pulmonary disease/COPD (J44), asthma (J45)), cancers (total (C00-C97, excluding non-melanoma skin cancer: C44), lung (C34), colorectal (C18-C20), oesophageal (C15), postmenopausal breast (C50) and prostate (C61)), neurological and mental disorders (total (F00-03, G12.2, G20, G21, G23.1-23.3, G23.8, G23.9, G30 and G31), Parkinson’s (G20), dementia including Alzheimer’s (A81.0, F00-02, F05.1, F10.6, G30, G31.0, G31.1 and G31.8), depression (F32-34, F38, F39) and anxiety (F40, F41), non-alcoholic fatty liver disease (NAFLD) (K74.0, K74.1, K74.2, K74.6, K75.8, K75.9, K76.0), chronic kidney disease (CKD) (ICD-10 codes: N03, N06, N08, N11-16, N18, N19, Z49, I12, I13 or Office of Population Censuses and Surveys Classification of Interventions and Procedures-version 4 (OPCS-4) codes in Hospital Inpatient data (OPCS-4 codes: L74.1-L74.6, L74.8, L74.9, M01.2, M01.3-M01.5, M01.8, M01.9, M02.3, M08.4, M17.2, M17.4, M17.8, M17.9, X40.1-X40.9, X41.1, X41.2, X41.8, X41.9, X42.1, X42.8, X42.9, and X43.1)), eczema/psoriasis (L20-27, L30, L40, L41), fracture (M484, M485, M800, M808, M809.0, M809.1-M809.3, M809.5-M809.9, M843, M844, S12, S22, S32, S42, S52, S72, S82, T02, T08, T10, T12) and osteoporosis (M80-82).

**Supplementary Methods 3** Multivariable adjustment

We used Cox proportional hazards models, with age (age-at-recruitment to age-at-event) as the time variable, to evaluate associations of the PDQS quartiles with mortality and morbidity outcomes. In models 1 we adjusted for sex and ethnicity; in models 2 we further adjusted for lifestyle (smoking, alcohol use, physical activity, total energy intake), health (use of blood thinners, use of nonsteroidal anti-inflammatory drugs, other disease such as cardiovascular disease and cancer) and sociodemographic (education, Townsend deprivation index) variables. Osteoporosis and fracture risk models were further adjusted for vitamin and mineral use. Finally, in models 3 we also adjusted for the baseline body mass index. The respiratory mortality and morbidity models 2 and 3 were also adjusted for smoking intensity and the pack years of smoking. Any cancer risk models were further adjusted for menopause status and hormone replacement therapy use in models 2 and 3. Prostate cancer models were limited only to men and breast cancer models only to postmenopausal women. All statistical models were stratified by region.

**Supplementary Methods 4 Missing data on covariates**

Missing values were handled by categorizing continuous variables (where appropriate) and recoding missing values as a ‘dummy missing variable’ for use in statistical models, hence preventing sample size reduction for each analysis.

The frequencies of missing values were as follows:

Ethnicity: 0.33%
Education level: 6.8%
Body mass index: 0.23%
Townsend deprivation score: 0.12%
Smoking status: 0.21%
Physical activity: 1.82%
Nonsteroidal anti-inflammatory drugs use: 14.6%
Hormone replacement therapy use: 0.12%
Vitamin and mineral supplement use: 0.03%
Menopausal status at recruitment: 0.02%
Ever use of oral contraception: 0.10%

**Supplementary Table S1** Comparison of baseline characteristics of the UK Biobank excluded participants (N=377,385) with included participants (N=124,851)

| **Characteristic** | **Excluded participants**  **No eligible dietary data** | **Included participants**  **Eligible dietary data available (≥2 diet recalls)** |
| --- | --- | --- |
|  | **Participants, No. (%)** | |
|  |  |  |
| Sex male, n (%) | 173,861 (46) | 55,151 (44) |
| Age at recruitment, years, mean (SD) | 56.7 (8) | 59 (8) |
| Ethnicity, n (%) |  |  |
| White | 351,843 (93) | 114,057 (91) |
| Mixed | 2,284 (1) | 3,449 (3) |
| Asian | 9,982 (3) | 5,752 (5) |
| Black | 7,086 (2) | 454 (0.5) |
| Townsend deprivation index, mean (SD) | -1.2 (3) | -1.6 (3) |
| Education level, n (%) |  |  |
| Low | 66,133 (18) | 31,990 (26) |
| Medium | 68,991 (18) | 19,972 (16) |
| High | 155,379 (41) | 64,396 (52) |
| Smoking status, n (%) |  |  |
| Never | 202,048 (54) | 71,340 (57) |
| Previous | 128,295 (34) | 44,660 (36) |
| Current | 44,360 (12) | 8,584 (7) |
| Alcohol use, g/day, mean (SD) | 20 (20) | 17 (20) |
| Physical activity, MET hr/wk, mean (SD) | 36 (52) | 31 (38) |
| BMI, mean (SD) | 28 (5) | 27 (5) |
| Cancer at baseline^a^, n (%) | 28,847 (8) | 10,119 (8) |
| CVD at baseline^a^, n (%) | 13,516 (4) | 4,676 (4) |
| T2D at baseline^a^, n (%) | 22,145 (6) | 4,839 (4) |

^a^ Either hospital in-patient or self-reported data from verbal interviews at baseline.
*Abbreviations: BMI, body mass index; MET, metabolic equivalent task; NSAID, nonsteroidal anti-inflammatory drugs; CVD, cardiovascular disease; T2D, type 2 diabetes*

| **Supplementary Table S2 Prime Diet Quality Score (PDQS) components and UK Biobank food items included** | | |
| --- | --- | --- |
|  | **UK Biobank food items included^a^** | **Scoring ranges**  **(g/day)** |
| ***Positively scored components*** |  |  |
| Dark green leafy vegetables | Celery, lettuce, spinach, watercress | <10/10-39/>39 |
| Cruciferous vegetables | Broccoli, coleslaw, cabbage kale, cauliflower, turnip, sprouts | <11/11-44/>44 |
| Deep orange vegetables | Butternut squash, carrot | <10/10-39/>39 |
| Deep orange fruits | Mango, melon | <28/28-114/>114 |
| Deep orange tubers | Sweet potato | <14/14-57/>57 |
| Other vegetables | Mixed vegetables, side salad, avocado, green bean, beetroot, courgette, cucumber, garlic, leek, mushroom, onion, parsnip, sweet pepper, tomato, tinned tomato | <26/26-106/>106 |
| Citrus fruits | Orange, grapefruit, satsuma | <18/18-74/>74 |
| Other fruits | Stewed fruits, prune, mixed fruits, apple, banana, berry, cherry, grape, peach nectarine, pear, pineapple, plum, other | <26/26-106/>106 |
| Legumes | Baked bean, pulses, broad bean, peas, tofu, soy milk | <10/10-39/>39 |
| Nuts and seeds | Salted nuts, unsalted nuts, salted peanuts, unsalted peanuts, seeds | <4/4-16/>16 |
| Poultry | Poultry, other meat | <12/12-48/>48 |
| Fish | Tinned tuna, oily fish, breaded fish, battered fish, white fish, other fish | <16/16-63/>63 |
| Wholegrains | Breads, baguette, bap and bread roll (only non-white: mixed, whole meal, seeded, other); crispbread, oatcakes, whole meal pasta, brown rice, other grain, sweet corn, porridge, muesli, oat crunch, bran crunch, other cereal | <4/4-16/>16 |
| Liquid oils | Type of oil in cooking (only liquid oils: olive, vegetable, sunflower, rapeseed, other oil) | If used then 2 points, otherwise 0. |
| Low fat dairy | Milk (only low fat: skimmed, semi, cholesterol lowering), cottage cheese, low fat cheese, low fat hard cheese, low fat yogurt | <35/35-139/>139 |
| ***Negatively scored components*** |  |  |
| White roots and tubers | Fried potatoes, boiled potato, mashed potato | <25/25-100/>100 |
| Red meat | Beef, pork, lamb, liver | <12/12-48/>48 |
| Processed meat | Sausage, bacon, ham | <8/8-31/>31 |
| Refined grains and baked goods | Breads, baguette, bap and bread roll (only white); white pasta, white rice, sushi, snack pot, couscous, naan, garlic bread, other bread, croissant, Danish pastry, scone, single crust pastry, double crust pastry, sweet cereal, plain cereal, savory biscuits, cheesy biscuits, other savory snack | <3.5/3.5-14/>14 |
| Sugar-sweetened beverages | Fizzy drink, squash | <52/52-207/>207 |
|  |  |  |
|  | **UK Biobank food items included^a^** | **Scoring ranges**  **(g/day)** |
| Sweets and ice cream | Pancake, scotch pancake, Yorkshire pudding, ice cream, milk-based pudding, other milk based pudding, soy dessert, fruit cake, cake, doughnut, sponge pudding, cheesecake, other dessert, chocolate bar, white chocolate, milk chocolate, chocolate covered raisin, chocolate sweets, other sweets, sweets, crumble, chocolate covered biscuit, chocolate biscuit, sweet biscuit, cereal bar | <11/11-45/>45 |
| Fried foods | Fried potatoes, fried poultry, breaded fish, battered fish, Indian snack | <10/10-40/>40 |
| ***Neutral components*** |  |  |
| Eggs | Eggs, whole egg, omelet, egg sandwich, scotch egg, other egg | Assign a constant^b^: 2 points. |

^a^ UK Biobank variables from 24-hour diet recall at follow-up.
^b^ Eggs are scored as a neutral component for adults.

| **Supplementary Table S3 Covariate coding and categorisation information** | | | | | | |
| --- | --- | --- | --- | --- | --- | --- |
| **Variables** | | | **Categorisation** | | | **UK Biobank variable description and data-field ID** |
| Demographics | | |  | | |  |
| Age | | | Age (years), continuous | | | Age at recruitment (ID: 21022)^a^ |
| Sex | | | Female; Male | | | Sex (ID: 31) ^a^ |
| Ethnicity | | | Asian, Black, Multiple, White, Other; Unknown/Missing | | | Ethnic background (ID: 21000) ^a^ |
| Socioeconomic status | | | | | |  |
| Education | | | Low: CSEs or equivalent, O levels/GCSEs or equivalent; Medium: A levels/AS levels or equivalent, NVQ or HND or HNC or equivalent; High: College or University degree, other professional qualifications e.g.: nursing, teaching; Unknown/Missing | | | Qualifications (ID: 6138) ^a^ |
| Townsend deprivation index | | | Quintiles from least to most deprived; Unknown/ Missing | | | Townsend deprivation index (ID: 189) ^a^ |
| Lifestyle and Environment | | | | | |  |
| Alcohol intake | | | <1g/d; 1-7g/d; 8-15g/d; 16+g/d; Unknown/Missing | | | Alcohol intake frequency (ID:1558) ^a^; Average weekly/monthly red wine intake (ID:1568/ID:4407) ^a^; Average weekly/monthly white wine intake (ID:1578/ID:4418)^a^; Average weekly/monthly beer intake (ID: 1588/ID:4429) ^a^; Average weekly/monthly spirits intake (ID: 4440/ ID:1598) ^a^; Average weekly/monthly fortified wine intake (ID:1608/ ID:4451) ^a^; Average weekly/monthly intake of other alcoholic drinks (ID: 5364/ ID:4462) ^a.^ |
| Smoking status | | | Never; Previous; Current; Unknown/Missing | | | Smoking status (ID: 20116) ^a^ |
| Smoking intensity | | | Never; Previous; Current <15/day; Current 15+/day; Current-intensity unknown; Missing | | | Smoking status (ID: 20116) ^a^; Number of cigarettes currently smoked daily (ID: 3456) ^a^ |
|  | | |  | | |  |
| Physical activity | | | METs hr/week quintiles; Unknown/Missing | | | Duration of walks (ID: 874) ^a^; Number of days/week walked 10+ minutes (ID: 864) ^a^; Duration of moderate activity (ID: 894) ^a^; Number of days/week of moderate physical activity 10+ minutes (ID: 884) ^a^; Duration of vigorous activity (ID: 914) ^a^; Number of days/week of vigorous physical activity 10+ minutes (ID: 904) ^a^ |
| Energy intake | | | Energy intake (kJ/day) (mean value across dietary assessments 1-5). | | | Energy (ID: 26002)^b^ |
| Health status | | |  | | |  |
| BMI | | | Underweight (<18.5 kg/m2); Healthy weight (18.5-24.99 kg/m2); Overweight (25-29.99 kg/m2); Obese (≥30 kg/m2); Unknown/Missing | | | BMI (ID: 21001)^c^ |
| **Variables** | | **Categorisation** | | | **UK Biobank variable description and data-field ID** | |
| CVD at baseline | | | No; Yes | | | Non cancer illness code, self-reported (ID: 20002)^a^; Diagnoses – ICD10 (ID: 41270)^d^; Date of first in-patient diagnosis – ICD10 (ID: 41280)^d^ |
| Type 2 diabetes at baseline | | | No; Yes | | | Non cancer illness code, self-reported (ID: 20002)^a^; Diagnoses – ICD10 (ID: 41270)^d^; Date of first in-patient diagnosis – ICD10 (ID: 41280)^d^ |
| Cancer at baseline | | | No; Yes | | | Non cancer illness code, self-reported (ID: 20002)^a^; Diagnoses – ICD10 (ID: 41270)^d^; Date of first in-patient diagnosis – ICD10 (ID: 41280)^d^ |
| Menopausal status | | | No; Yes; Not sure (hysterectomy/other reason); Men | | | Had menopause (women only) (ID: 2724)^a^ |
| HRT use | | | No; Yes; Unknown/Missing | | | Ever used hormone-replacement therapy (HRT) (ID:2814)^a^ |
| Blood thinning medication | | | No; Yes; Unknown/Missing | | | Medication for pain relief, constipation, heartburn (ID: 6154)^a^; Treatment/medication code (ID: 20003)^e^ |
| NSAIDs | | | No; Yes; Uknown/Missing | | | Medication for pain relief, constipation, heartburn (ID: 6154)^a^; Treatment/medication code (ID: 20003)^e^ |
| **Variables** | **Categorisation** | | | **UK Biobank variable description and data-field ID** | | |
| Vitamin and mineral supplement use | | | Vitamin A; Vitamin; Vitamin C; Vitamin D; Vitamin E; Folic acid or folate (Vitamin B9); Multivitamins +/- minerals; Unknown/Missing | | | Vitamin and mineral supplements (ID: 6155)^a^ |
| PRS (Alzheimer’s disease) [4] | | | Tertiles from low to high PRS for Alzheimer’s disease; Unknown/Missing | | | Standard PRS for Alzheimer’s disease (ID: 26206)^f^ |
| PRS (ischemic stroke) [4] | | | Tertiles from low to high PRS for ischemic stroke; Unknown/Missing | | | Standard PRS for ischemic stroke (ID: 26248)^f^ |
| ^a^ Data collected at recruitment via touchscreen questionnaire (initial assessment visit (2006-2010). ^b^ Data collected from 24-hr online Oxford WebQ dietary questionnaire (assessment centre (April 2009 to September 2010; on-line cycle 1 (February 2011 to April 2011); on-line cycle 2 (June 2011 to September 2011); on-line cycle 3 (October 2011 to December 2011); on-line cycle 4 (April 2012 to June 2012). ^c^ Physical measurements (initial assessment visit (2006-2010). ^d^ Hospital inpatient admission data. ^e^ Data collected via verbal interview (initial assessment visit (2006-2010). ^f^ Genomics data (using blood samples from initial assessment visit (2006-2010). *Abbreviations: BMI, body mass index; MET, metabolic equivalent task; NSAID, nonsteroidal anti-inflammatory drugs; CVD, cardiovascular disease; HRT, hormone replacement therapy; PRS, polygenic risk score.* | | | | | | |

**Supplementary Table S4 Intraclass correlation coefficients (ICCs) for reproducibility of the Prime Diet Quality Score (PDQS) over time**

| **24-hour dietary recall cycles** | **T1** | **T2** | **T3** | **T4** | **T5** | **T4+T5** |
| --- | --- | --- | --- | --- | --- | --- |
| T1 | - | 0.89 (0.89, 0.89) | 0.90 (0.90, 0.90) | 0.89 (0.89, 0.89) | 0.89 (0.89, 0.89) | - |
| T2 |  | - | 0.82 (0.82, 0.82) | 0.80 (0.80, 0.80) | 0.80 (0.80, 0.80) | - |
| T3 |  |  | - | 0.80 (0.80, 0.80) | 0.81 (0.81, 0.81) | - |
| T4 |  |  |  | - | 0.78 (0.78, 0.79) | - |
| T5 |  |  |  |  | - | - |
| T2+T3 |  |  |  |  | *-* | 0.78 (0.77, 0.78) |
| T4+T5 |  |  |  |  | *-* | - |

*Abbreviations: T1, first 24-hour diet recall; T2, second 24-hour diet recall; T3, third 24-hour diet recall: T4, fourth 24-hour diet recall; T5, fifth 24-hour diet recall, T2+T3, average of the
second and the third 24-hour diet recall, T4+T5, average of the fourth and the fifth 24-hour diet recall.*

**Supplementary Table S5** **Hazard ratios and 95% confidence intervals between the Prime Diet Quality Score (PDQS) and mortality in UK Biobank^a^**

|  | **Q1** | **Q2** | **Q3** | **Q4** | **P-trend** | **Corrected P-trend^b^** |
| --- | --- | --- | --- | --- | --- | --- |
| *Person-years* | 355710.1 | 217538.4 | 296381.2 | 244392.6 |  |  |
| *All-cause mortality* |  |  |  |  |  |  |
| Cases/participants | 1,404/33,750 | 773/20,633 | 923/28,114 | 742/23,257 |  |  |
| Model 1^c^ | 1.00 (ref) | 0.87 (0.80, 0.95) | 0.75 (0.69, 0.81) | 0.72 (0.66, 0.79) | <.0001 |  |
| Model 2^d^ | 1.00 (ref) | 0.91 (0.83, 1.00) | 0.80 (0.73, 0.87) | 0.79 (0.72, 0.86) | <.0001 |  |
| Model 3^e^ | 1.00 (ref) | 0.92 (0.84, 1.01) | 0.81 (0.74, 0.88) | **0.80 (0.73, 0.88)** | **<.0001** | .0005 |
| *Cancer mortality* |  |  |  |  |  |  |
| Cases/participants | 784/33,750 | 474/20,663 | 537/28,114 | 463/23,257 |  |  |
| Model 1^c^ | 1.00 (ref) | 0.95 (0.84, 1.06) | 0.77 (0.69, 0.86) | 0.79 (0.70, 0.89) | <.0001 |  |
| Model 2^d^ | 1.00 (ref) | 0.99 (0.88, 1.11) | 0.81 (0.73, 0.91) | 0.85 (0.76, 0.96) | <.0001 |  |
| Model 3^e^ | 1.00 (ref) | 1.00 (0.89, 1.12) | 0.82 (0.74, 0.92) | **0.87 (0.77, 0.98)** | **.003** | .008 |
| *CVD mortality* |  |  |  |  |  |  |
| Cases/participants | 165/33,750 | 94/20,663 | 108/28,114 | 108/23,257 |  |  |
| Model 1^c^ | 1.00 (ref) | 0.90 (0.70, 1.17) | 0.75 (0.59, 0.96) | 0.91 (0.71, 1.17) | .14 |  |
| Model 2^d^ | 1.00 (ref) | 0.95 (0.73, 1.22) | 0.80 (0.62, 1.02) | 0.98 (0.62, 1.02) | .43 |  |
| Model 3^e^ | 1.00 (ref) | 0.96 (0.74, 1.24) | 0.81 (0.63, 1.04) | 1.01 (0.79, 1.31) | .58 | .58 |
| *Respiratory mortality* |  |  |  |  |  |  |
| Cases/participants | 99/33,750 | 30/20,663 | 38/28,114 | 29/23,257 |  |  |
| Model 1^c^ | 1.00 (ref) | 0.48 (0.31, 0.71) | 0.43 (0.30, 0.63) | 0.40 (0.26, 0.61) | <.0001 |  |
| Model 2 ^d,f^ | 1.00 (ref) | 0.58 (0.38, 0.87) | 0.58 (0.38, 0.85) | 0.57 (0.37, 0.87) | .006 |  |
| Model 3^e,f^ | 1.00 (ref) | 0.59 (0.31, 0.90) | 0.59 (0.40, 0.86) | **0.56 (0.37, 0.87)** | **.007** | .01 |
| *Neurodegenerative mortality* |  |  |  |  |  |  |
| Cases/participants | 62/33,750 | 36/20,663 | 48/28,114 | 29/23,257 |  |  |
| Model 1^c^ | 1.00 (ref) | 0.90 (0.60, 1.35) | 0.84 (0.58, 1.24) | 0.62 (0.40, 0.97) | .04 |  |
| Model 2^d^ | 1.00 (ref) | 0.91 (0.60, 1.37) | 0.86 (0.58, 1.26) | 0.62 (0.39, 0.97) | .04 |  |
| Model 3^e^ | 1.00 (ref) | 0.90 (0.59, 1.35) | 0.85 (0.58, 1.24) | **0.61 (0.39, 0.96)** | **.04** | .05 |

^a^ N=105,754. ^b^ Corrected for multiple comparisons using the Benjamini-Hochberg procedure.
^c^ Adjusted for sex and ethnicity, stratified by region.
^d^ Also adjusted for education category, social deprivation index (in quintiles), smoking status (never, current, past), alcohol consumption, physical activity quintile, use of blood thinning medications, use of NSAID, TEI (KJ/day).
^e^ Also adjusted for BMI.
^f^ Also adjusted for smoking intensity (number of cigarettes currently smoked/day) and pack years of smoking.

*Abbreviations: BMI, body mass index; NSAID, nonsteroidal anti-inflammatory drugs; TEI, total energy intake.*

**Supplementary Table S6 Hazard ratios and 95% confidence intervals between the Prime Diet Quality Score (PDQS) and cardiometabolic diseases in UK Biobank^a^**

|  | **Q1** | **Q2** | **Q3** | **Q4** | **P-trend** | **Corrected P-trend^b^** |
| --- | --- | --- | --- | --- | --- | --- |
| *T2D* |  |  |  |  |  |  |
| *Person-years* | 389078.2 | 239282.1 | 328437.2 | 273094.2 |  |  |
| Cases/participants | 1,413/37,978 | 681/23,262 | 777/31,864 | 550/26,541 |  |  |
| Model 1^c^ | 1.00 (ref) | 0.78 (0.72, 0.86) | 0.65 (0.60, 0.71) | 0.56 (0.51, 0.62) | <.0001 |  |
| Model 2^d,e^ | 1.00 (ref) | 0.84 (0.77, 0.92) | 0.72 (0.66, 0.79) | 0.64 (0.58, 0.71) | <.0001 |  |
| Model 3^f^ | 1.00 (ref) | 0.90 (0.82, 0.99) | 0.78 (0.72, 0.86) | **0.73 (0.66, 0.81)** | <.0001 | .0002 |
| *CVD, any* |  |  |  |  |  |  |
| *Person-years* | 385756.0 | 235980.0 | 324432.3 | 269223.8 |  |  |
| Cases/participants | 2,200/37,973 | 1,186/23,136 | 1,538/31,747 | 1,222/26,396 |  |  |
| Model 1^c^ | 1.00 (ref) | 0.88 (0.82, 0.94) | 0.83 (0.77, 0.88) | 0.81 (0.76, 0.87) | <.0001 |  |
| Model 2^d,e^ | 1.00 (ref) | 0.90 (0.84, 0.97) | 0.86 (0.81, 0.92) | 0.86 (0.81, 0.93) | <.0001 |  |
| Model 3^f^ | 1.00 (ref) | 0.91 (0.85, 0.98) | 0.87 (0.82, 0.93) | **0.88 (0.82, 0.95)** | <.0001 | .0002 |
| *Myocardial infarction* |  |  |  |  |  |  |
| Cases/participants | 918/37,973 | 493/23,136 | 599/31,747 | 427/26,396 |  |  |
| Model 1^c^ | 1.00 (ref) | 0.90 (0.81, 1.01) | 0.81 (0.73, 0.90) | 0.73 (0.65, 0.82) | <.0001 |  |
| Model 2^d,e^ | 1.00 (ref) | 0.94 (0.84, 1.05) | 0.86 (0.77, 0.95) | 0.78 (0.70, 0.88) | <.0001 |  |
| Model 3^f^ | 1.00 (ref) | 0.95 (0.85, 1.06) | 0.87 (0.78, 0.97) | **0.80 (0.71, 0.90)** | <.0001 | .0002 |
| *Stroke, any* |  |  |  |  |  |  |
| Cases/participants | 614/37,973 | 348/23,136 | 417/31,747 | 398/26,396 |  |  |
| Model 1^c^ | 1.00 (ref) | 0.87 (0.76, 1.00) | 0.75 (0.66, 0.85) | 0.87 (0.76, 0.99) | .01 |  |
| Model 2^d,e^ | 1.00 (ref) | 0.89 (0.78, 1.02) | 0.77 (0.68, 0.88) | 0.90 (0.79, 1.02) | .06 |  |
| Model 3^f^ | 1.00 (ref) | 0.99 (0.78, 1.02) | **0.78 (0.69, 0.88)** | 0.91 (0.80, 1.04) | .10 | .12 |
| *Ischemic stroke* |  |  |  |  |  |  |
| Cases/participants | 451/37973 | 244/23,136 | 291/31,747 | 271/26,396 |  |  |
| Model 1^c^ | 1.00 (ref) | 0.85 (0.73, 0.99) | 0.72 (0.62, 0.84) | 0.82 (0.70, 0.84) | .003 |  |
| Model 2^d,e^ | 1.00 (ref) | 0.86 (0.73, 1.01) | 0.74 (0.64, 0.86) | 0.85 (0.73, 0.99) | .02 |  |
| Model 3^f^ | 1.00 (ref) | 0.86 (0.74, 1.01) | 0.75 (0.64, 0.87) | **0.86 (0.74, 1.00)** | .04 | .06 |
| *Hemorrhagic stroke* |  |  |  |  |  |  |
| Cases/participants | 149/37,973 | 92/23,136 | 108/31,747 | 95/26,396 |  |  |
| Model 1^c^ | 1.00 (ref) | 0.92 (0.70, 1.19) | 0.77 (0.60, 0.99) | 0.78 (0.60, 1.02) | .03 |  |
| Model 2^d,e^ | 1.00 (ref) | 0.95 (0.73, 1.24) | 0.81 (0.63, 1.04) | 0.84 (0.64, 1.10) | .11 |  |
| Model 3^f^ | 1.00 (ref) | 0.95 (0.73, 1.24) | 0.81 (0.63, 1.04) | 0.84 (0.64, 1.10) | .14 | .14 |

^a^ N=119,252 (CVD), 119,645 (diabetes).
^b^ Corrected for multiple comparisons across all morbidity outcomes from one category using the Benjamini-Hochberg procedure.
^c^ Adjusted for sex and ethnicity, stratified by region.
^d^ Also adjusted for education category, social deprivation index, smoking status, alcohol consumption, physical activity quintile, use of blood thinning medications, use of NSAID, TEI (KJ/day).
^e^ Diabetes models also adjusted for CVD and cancer at baseline; CVD models also adjusted for T2D and cancer at baseline.
^f^ Also adjusted for BMI.  *Abbreviations: BMI, body mass index; NSAID, nonsteroidal anti-inflammatory drugs; CVD, cardiovascular disease; T2D, type 2 diabetes; TEI, total energy intake.*

**Supplementary Table S7 Hazard ratios and 95% confidence intervals between the Prime Diet Quality Score (PDQS) and chronic respiratory outcomes in UK Biobank^a^**

|  | **Q1** | **Q2** | **Q3** | **Q4** | **P-trend** | **Corrected P-trend^b^** |
| --- | --- | --- | --- | --- | --- | --- |
| *Respiratory disease, any chronic* |  |  |  |  |  |  |
| *Person-years* | 334206.45 | 204898.31 | 283734.49 | 234941.95 |  |  |
| Cases/participants | 3,588/ 33,398 | 1,999/ 20,416 | 2,639/ 28,171 | 2,087/ 23,385 |  |  |
| Model 1^c^ | 1.00 (ref) | 0.88 (0.83, 0.93) | 0.82 (0.78, 0.86) | 0.78 (0.73, 0.82) | <.0001 |  |
| Model 2^d^ | 1.00 (ref) | 0.94 (0.89, 1.00) | 0.90 (0.86, 0.95) | 0.87 (0.86, 0.95) | <.0001 |  |
| Model 3^e^ | 1.00 (ref) | 0.95 (0.90, 1.00) | 0.91 (0.86, 0.96) | **0.89 (0.84, 0.94)** | <.0001 | .0003 |
| *COPD* |  |  |  |  |  |  |
| Cases/participants | 645/33,398 | 270/20,416 | 330/28,171 | 245/23,385 |  |  |
| Model 1^c^ | 1.00 (ref) | 0.66 (0.57, 0.76) | 0.57 (0.50, 0.65) | 0.50 (0.43, 0.59) | <.0001 |  |
| Model 2^d^ | 1.00 (ref) | 0.85 (0.73, 0.98) | 0.81 (0.70, 0.93) | 0.81 (0.69, 0.95) | .002 |  |
| Model 3^e^ | 1.00 (ref) | 0.85 (0.74, 0.99) | 0.81 (0.71, 0.93) | **0.80 (0.69, 0.94)** | .001 | .002 |
| *Asthma* |  |  |  |  |  |  |
| Cases/participants | 720/33,398 | 383/20,416 | 521/28,171 | 442/23,385 |  |  |
| Model 1^c^ | 1.00 (ref) | 0.83 (0.74, 0.94) | 0.80 (0.71, 0.90) | 0.80 (0.71, 0.90) | <.0001 |  |
| Model 2^d^ | 1.00 (ref) | 0.87 (0.76, 0.98) | 0.84 (0.75, 0.95) | **0.85 (0.76, 0.97)** | .01 |  |
| Model 3^e^ | 1.00 (ref) | 0.89 (0.78, 1.00) | **0.87 (0.78, 0.98)** | 0.89 (0.79, 1.01) | .08 | .08 |

^a^ N=105,370.
^b^ Corrected for multiple comparisons across all morbidity outcomes from one category using the Benjamini-Hochberg procedure.
^c^ Adjusted for sex and ethnicity, stratified by region.
^d^ Also adjusted for education category, social deprivation index, smoking status, smoking intensity, pack years of smoking, alcohol consumption, physical activity quintile, use of blood thinning medications, use of NSAID, cancer, CVD and T2D at baseline, TEI (KJ/day).
^e^ Also adjusted for BMI.
*Abbreviations: BMI, body mass index; NSAID, nonsteroidal anti-inflammatory drugs; CVD, cardiovascular disease; T2D, type 2 diabetes; COPD, chronic obstructive pulmonary disease; TEI, total energy intake.*

**Supplementary Table S8 Hazard ratios and 95% confidence intervals between the Prime Diet Quality Score (PDQS) and COPD across smoking status categories in UK Biobank**

| ***COPD*** | **Q1** | **Q2** | **Q3** | **Q4** | **P-trend** | **P-interaction** |
| --- | --- | --- | --- | --- | --- | --- |
| Cases/participants | 84/18,737 | 59/11,773 | 68/16,432 | 50/13,808 |  |  |
| Never smokers^a^ | 1.00 (ref) | 1.12 (0.80, 1.57) | 0.90 (0.64, 1.24) | 0.78 (0.54, 1.12) | .08 | .52 |
| Cases/participants | 298/11,362 | 127/7,238 | 190/10,085 | 142/8,500 |  |  |
| Past smokers^a^ | 1.00 (ref) | 0.78 (0.63, 0.97) | 0.84 (0.70, 1.01) | 0.84 (0.68, 1.03) | .05 |  |
| Cases/participants | 261/3,217 | 82/1,363 | 72/1,595 | 53/1,032 |  |  |
| Current smokers^a^ | 1.00 (ref) | 0.87 (0.67, 1.12) | 0.72 (0.55, 0.94) | 0.84 (0.62, 1.15) | .20 |  |
|  |  |  |  |  |  |  |
| Cases/participants | 559/14,579 | 209/8,601 | 262/11,680 | 195/9,532 |  |  |
| Never+past smokers^a^ | 1.00 (ref) | 0.80 (0.68, 0.95) | 0.80 (0.69, 0.94) | **0.82 (0.69, 0.97)** | .01 |  |

^a^Adjusted for sex and ethnicity, education category, social deprivation index, smoking status, smoking intensity, pack years of smoking (smoking variables: except among never smokers), alcohol consumption, physical activity quintile, use of blood thinning medications, use of NSAID, cancer, CVD and T2D at baseline, TEI (KJ/day) and BMI; stratified by region.
*Abbreviations: BMI, body mass index; NSAID, nonsteroidal anti-inflammatory drugs; CVD, cardiovascular disease; T2D, type 2 diabetes; COPD, chronic obstructive pulmonary disease; TEI, total energy intake.*

**Supplementary Table S9 Hazard ratios and 95% confidence intervals between the Prime Diet Quality Score (PDQS) and cancer in UK Biobank^a^**

|  | **Q1** | **Q2** | **Q3** | **Q4** | **P-trend** | **Corrected P-trend^b^** |
| --- | --- | --- | --- | --- | --- | --- |
| *Cancer, any* |  |  |  |  |  |  |
| *Person-years* | 301927.35 | 182702.25 | 247463.05 | 202610.34 |  |  |
| Cases/participants | 3,125/36,236 | 1,926/21,932 | 2,418/29,687 | 1,969/24,366 |  |  |
| Model 1^c^ | 1.00 (ref) | 0.99 (0.94, 1.05) | 0.90 (0.86, 0.95) | 0.89 (0.84, 0.95) | <.0001 |  |
| Model 2^d^ | 1.00 (ref) | 1.01 (0.95,1.06) | 0.92 (0.87, 0.97) | 0.93 (0.87, 0.98) | .003 |  |
| Model 3^e^ | 1.00 (ref) | 1.01 (0.95, 1.07) | 0.93 (0.88, 0.98) | **0.94 (0.88, 0.99)** | .01 | .03 |
| *Lung cancer* |  |  |  |  |  |  |
| Cases/participants | 212/36,236 | 111/21,932 | 123/29,687 | 90/24,366 |  |  |
| Model 1^c^ | 1.00 (ref) | 0.80 (0.64, 1.01) | 0.63 (0.50, 0.78) | 0.53 (0.41, 0.68) | <.0001 |  |
| Model 2^d,f^ | 1.00 (ref) | 0.98 (0.78, 1.24) | 0.82 (0.65, 1.03) | 0.76 (0.59, 0.98) | .005 |  |
| Model 3^e,f^ | 1.00 (ref) | 0.99 (0.78, 1.25) | 0.82 (0.65, 1.03) | **0.75 (0.58, 0.97)** | .004 | .02 |
| *Colorectal cancer* |  |  |  |  |  |  |
| Cases/participants | 348/36,236 | 220/21,932 | 254/29,687 | 220/24,366 |  |  |
| Model 1^c^ | 1.00 (ref) | 1.03 (0.87, 1.21) | 0.87 (0.74, 1.02) | 0.92 (0.78, 1.10) | .08 |  |
| Model 2^d^ | 1.00 (ref) | 1.05 (0.89, 1.25) | 0.90 (0.76, 1.06) | 0.98 (0.83, 1.17) | .31 |  |
| Model 3^e^ | 1.00 (ref) | 1.06 (0.89, 1.26) | 0.91 (0.77, 1.07) | 1.00 (0.84, 1.20) | .45 | .68 |
| *Oesophageal cancer* |  |  |  |  |  |  |
| Cases/participants | 67/36,236 | 40/21,932 | 51/29,687 | 27/24,366 |  |  |
| Model 1^c^ | 1.00 (ref) | 0.97 (0.66, 1.44) | 0.92 (0.64, 1.33) | **0.62 (0.40, 0.98)** | .04 |  |
| Model 2^d^ | 1.00 (ref) | 1.05 (0.71, 1.56) | 1.02 (0.71, 1.48) | 0.74 (0.46, 1.16) | .23 |  |
| Model 3^e^ | 1.00 (ref) | 1.09 (0.73, 1.61) | 1.06 (0.73, 1.54) | 0.77 (0.49, 1.23) | .34 | .68 |
| *Prostate cancer^g^* |  |  |  |  |  |  |
| Cases/participants | 815/20,171 | 474/10,173 | 577/12,248 | 388/7,913 |  |  |
| Model 1^c^ | 1.00 (ref) | 1.06 (0.95, 1.19) | 1.02 (0.92, 1.14) | 1.02 (0.91, 1.15) | .59 |  |
| Model 2^d^ | 1.00 (ref) | 1.05 (0.94, 1.17) | 1.01 (0.90, 1.12) | 1.00 (0.88, 1.13) | .95 |  |
| Model 3^e^ | 1.00 (ref) | 1.05 (0.99, 1.17) | 1.00 (0.90, 1.12) | 1.00 (0.88, 1.13) | .99 | .99 |
| *Postmenopausal breast cancer^h^* |  |  |  |  |  |  |
| Cases/participants | 318/10,699 | 295/8,400 | 382/12,827 | 372/12,764 |  |  |
| Model 1^c^ | 1.00 (ref) | 1.17 (1.00, 1.37) | 0.98 (0.85, 1.14) | 0.96 (0.82, 1.11) | .39 |  |
| Model 2^d^ | 1.00 (ref) | 1.19 (1.04, 1.40) | 1.00 (0.86, 1.17) | 0.99 (0.85, 1.16) | .69 |  |
| Model 3^e^ | 1.00 (ref) | 1.20 (1.02, 1.41) | 1.02 (0.88, 1.18) | 1.02 (0.87, 1.18) | .97 | .99 |

^a^ N=112,221 (all cancer), 50,503 (prostate cancer), 44,690 (breast cancer).
^b^ Corrected for multiple comparisons across all morbidity outcomes from one category using the Benjamini-Hochberg procedure.
^b^ Adjusted for sex and ethnicity, stratified by region.
^d^ Also adjusted for education category, social deprivation index, smoking status, alcohol consumption category, physical activity quintile, CVD or T2D at baseline, use of blood thinning medications, menopause status at baseline (any cancer analysis, women only), use of HRT (any cancer analysis, women only), use of NSAID, TEI (KJ/day).
^e^ Also adjusted for BMI.
^f^ Also adjusted for smoking intensity (number of cigarettes currently smoked) and pack years of smoking.
^g^ Analysis restricted only to men (N=50,505).
^h^ Analysis restricted only to postmenopausal women (N=44,690).
*Abbreviations: BMI, body mass index; NSAID, nonsteroidal anti-inflammatory drugs; CVD, cardiovascular disease; T2D, type 2 diabetes; TEI, total energy intake; HRT, hormone replacement therapy.*

**Supplementary Table S10 Hazard ratios and 95% confidence intervals between the Prime Diet Quality Score (PDQS) and neurological and psychological disorders in UK Biobank^a^**

|  | **Q1** | **Q2** | **Q3** | **Q4** | **P-trend** | **Corrected P-trend^b^** |
| --- | --- | --- | --- | --- | --- | --- |
| *Neurodegenerative disease, total* |  |  |  |  |  |  |
| *Person-years* | 412154.0 | 250309.6 | 342803.0 | 283254.3 |  |  |
| Cases/participants | 613/39,837 | 384/24,179 | 477/33,068 | 447/27,428 |  |  |
| Model 1^c^ | 1.00 (ref) | 0.95 (0.83, 1.07) | 0.83 (0.73, 0.93) | 0.92 (0.81, 1.04) | .05 |  |
| Model 2^d^ | 1.00 (ref) | 0.97 (0.85, 1.10) | 0.85 (0.75, 0.96) | 0.96 (0.85, 1.09) | .25 |  |
| Model 3^e^ | 1.00 (ref) | 0.97 (0.85, 1.10) | **0.85 (0.75, 0.96)** | 0.96 (0.85, 1.09) | .24 | .79 |
| *Parkinson’s disease* |  |  |  |  |  |  |
| *Person-years* | 413371.4 | 251077.4 | 343663.4 | 284086.4 |  |  |
| Cases/participants | 204/39,879 | 126/24,207 | 162/33,092 | 151/27,454 |  |  |
| Model 1^c^ | 1.00 (ref) | 0.95 (0.76, 1.18) | 0.87 (0.71, 1.07) | 0.98 (0.79, 1.22) | .56 |  |
| Model 2^d^ | 1.00 (ref) | 0.95 (0.76, 1.19) | 0.88 (0.71, 1.08) | 1.00 (0.81, 1.24) | .67 |  |
| Model 3^e^ | 1.00 (ref) | 0.95 (0.76, 1.19) | 0.87 (0.71, 1.08) | 1.00 (0.80, 1.24) | .65 | .79 |
| *Dementia, any* |  |  |  |  |  |  |
| *Person-years* | 413925.8 | 251366.3 | 344122.0 | 284253.9 |  |  |
| Cases/participants | 273/39,933 | 181/24,238 | 236/33,136 | 235/27,464 |  |  |
| Model 1^c^ | 1.00 (ref) | 0.98 (0.81, 1.19) | 0.90 (0.75, 1.07) | 1.06 (0.88, 1.26) | .91 |  |
| Model 2^d^ | 1.00 (ref) | 1.00 (0.83, 1.21) | 0.92 (0.77, 1.10) | 1.09 (0.91, 1.30) | .60 |  |
| Model 3^e^ | 1.00 (ref) | 1.01 (0.83, 1.21) | 0.92 (0.77, 1.10) | 1.08 (0.90, 1.30) | .64 | .79 |
| *Alzheimer’s dementia* |  |  |  |  |  |  |
| Cases/participants | 130/39,933 | 119/24,238 | 123/33,136 | 124/27,464 |  |  |
| Model 1^c^ | 1.00 (ref) | 1.33 (1.04, 1.71) | 0.95 (0.74, 1.22) | 1.10 (0.86, 1.42) | .81 |  |
| Model 2^d^ | 1.00 (ref) | 1.34 (1.04 1.72) | 0.96 (0.75, 1.23) | 1.12 (0.87, 1.45) | .67 |  |
| Model 3^e^ | 1.00 (ref) | 1.33 (1.04, 1.71) | 0.95 (0.74, 1.23) | 1.10 (0.86, 1.43) | .79 | .79 |
| *Depression* |  |  |  |  |  |  |
| *Person-years* | 384975.0 | 236198.6 | 323867.1 | 267495.4 |  |  |
| Cases/participants | 1108/37,509 | 566/22,975 | 763/31,448 | 693/26,076 |  |  |
| Model 1^c^ | 1.00 (ref) | 0.80 (0.72, 0.88) | 0.76 (0.70, 0.83) | 0.80 (0.73, 0.88) | <.0001 |  |
| Model 2^d^ | 1.00 (ref) | 0.85 (0.77, 0.95) | 0.83 (0.76, 0.92) | **0.90 (0.82, 1.00)** | .002 |  |
| Model 3^e^ | 1.00 (ref) | 0.87 (0.79, 0.97) | **0.86 (0.78, 0.94)** | 0.93 (0.85, 1.03) | .02 | .02 |
| *Anxiety* |  |  |  |  |  |  |
| *Person-years* | 402685.78 | 245339.25 | 335412.67 | 276924.05 |  |  |
| Cases/participants | 1452/39,330 | 801/23,912 | 1065/32,650 | 926/27,043 |  |  |
| Model 1^c^ | 1.00 (ref) | 0.83 (0.76, 0.91) | 0.77 (0.71, 0.83) | 0.75 (0.69, 0.82) | <.0001 |  |
| Model 2^d^ | 1.00 (ref) | 0.88 (0.81, 0.96) | 0.83 (0.76, 0.90) | 0.83 (0.77, 0.91) | <.0001 |  |
| Model 3^e^ | 1.00 (ref) | 0.89 (0.82, 0.97) | 0.84 (0.78, 0.91) | **0.85 (0.78, 0.92)** | .0003 | .006 |

^a^ N=124,511 (neurodegenerative disease), 124,632 (Parkinson’s), 124,770 (dementia), 118,007 (depression), 122,935 (anxiety).
^b^ Corrected for multiple comparisons across all morbidity outcomes from one category using the Benjamini-Hochberg procedure.
^c^ Adjusted for sex and ethnicity, stratified by region.
^d^ Also adjusted for education category, social deprivation index, smoking status, alcohol consumption category, physical activity quintile, CVD,
cancer or T2D at baseline, use of blood thinning medications, use of NSAID, TEI (KJ/day).
^e^ Also adjusted for BMI.
*Abbreviations: BMI, body mass index; NSAID, nonsteroidal anti-inflammatory drugs; CVD, cardiovascular disease; T2D, type 2 diabetes; TEI, total energy intake.*

**Supplementary Table S11 Hazard ratios and 95% confidence intervals between the Prime Diet Quality Score (PDQS) and other chronic diseases in UK Biobank^a^**

|  | **Q1** | **Q2** | **Q3** | **Q4** | **P-trend** | **Corrected P-trend^b^** |
| --- | --- | --- | --- | --- | --- | --- |
| *Nonalcoholic fatty liver disease* |  |  |  |  |  |  |
| *Person-years* | 410765.3 | 250100.2 | 342645.4 | 283265.7 |  |  |
| Cases/participants | 572/39,757 | 292/24,177 | 323/33,045 | 210/27,396 |  |  |
| Model 1^c^ | 1.00 (ref) | 0.82 (0.71, 0.95) | 0.65 (0.57, 0.95) | 0.50 (0.43, 0.59) | <.0001 |  |
| Model 2^d^ | 1.00 (ref) | 0.89 (0.77, 1.02) | 0.72 (0.63, 0.83) | 0.59 (0.50, 0.70) | <.0001 |  |
| Model 3^e^ | 1.00 (ref) | 0.94 (0.82, 1.09) | 0.79 (0.68, 0.90) | **0.66 (0.56, 0.77)** | <.0001 | .0003 |
| *Chronic kidney disease* |  |  |  |  |  |  |
| *Person-years* | 404228.5 | 246250.9 | 337710.4 | 279650.7 |  |  |
| Cases/participants | 2063/39,572 | 1096/24,048 | 1362/32,887 | 1038/27,315 |  |  |
| Model 1^c^ | 1.00 (ref) | 0.82 (0.76, 0.88) | 0.72 (0.67, 0.77) | 0.65 (0.60, 0.70) | <.0001 |  |
| Model 2^d^ | 1.00 (ref) | 0.86 (0.80, 0.92) | 0.76 (0.71, 0.82) | 0.71 (0.65, 0.76) | <.0001 |  |
| Model 3^e^ | 1.00 (ref) | 0.88 (0.82, 0.95) | 0.79 (0.73, 0.84) | **0.74 (0.69, 0.80)** | <.0001 | .0003 |
| *Eczema/psoriasis* |  |  |  |  |  |  |
| *Person-years* | 394542.8 | 240362.0 | 329207.1 | 272265.3 |  |  |
| Cases/participants | 600/38,207 | 332/23,253 | 455/31,803 | 392/26,402 |  |  |
| Model 1^c^ | 1.00 (ref) | 0.88 (0.77, 1.02) | 0.86 (0.76, 0.97) | **0.87 (0.77, 0.99)** | .02 |  |
| Model 2^d^ | 1.00 (ref) | 0.91 (0.79, 1.04) | 0.90 (0.79, 1.01) | 0.93 (0.82, 1.06) | .25 |  |
| Model 3^e^ | 1.00 (ref) | 0.92 (0.80, 1.05) | 0.91 (0.81, 1.03) | 0.95 (0.84, 1.09) | .48 | .74 |
| *Fracture* |  |  |  |  |  |  |
| *Person-years* | 387569.4 | 234576.1 | 320533.9 | 264755.4 |  |  |
| Cases/participants | 1536/37,979 | 981/22,993 | 1425/31,444 | 1305/26,093 |  |  |
| Model 1^c^ | 1.00 (ref) | 0.96 (0.89, 1.04) | 0.97 (0.90, 1.04) | 1.00 (0.93, 1.08) | .99 |  |
| Model 2^d,f^ | 1.00 (ref) | 0.97 (0.89, 1.05) | 0.97 (0.91, 1.05) | 1.00 (0.93, 1.08) | .99 |  |
| Model 3^e,f^ | 1.00 (ref) | 0.96 (0.89, 1.04) | 0.96 (0.90, 1.04) | 0.98 (0.91, 1.06) | .60 | .74 |
| *Osteoporosis* |  |  |  |  |  |  |
| *Person-years* | 406046.41 | 245581.37 | 334720.74 | 274596.35 |  |  |
| Cases/participants | 727/39,372 | 553/23,830 | 878/32,492 | 821/26,778 |  |  |
| Model 1^c^ | 1.00 (ref) | 1.01 (0.91, 1.13) | 1.05 (0.95, 1.16) | 1.02 (0.93, 1.13) | .74 |  |
| Model 2^d,f^ | 1.00 (ref) | 1.04 (0.93, 1.16) | 1.08 (0.97, 1.19) | 1.06 (0.95, 1.17) | .35 |  |
| Model 3^e,f^ | 1.00 (ref) | 1.01 (0.90, 1.13) | 1.03 (0.94, 1.14) | 0.99 (0.89, 1.10) | .74 | .74 |

^a^ N= 124,374 (nonalcoholic fatty liver disease), 123,822 (chronic kidney disease), 119,665 (eczema/psoriasis), 118,509 (fracture), 122,472 (osteoporosis). ^b^ Corrected for multiple comparisons across all morbidity outcomes from one category using the Benjamini-Hochberg procedure.
^c^ Adjusted for sex and ethnicity, stratified by region.
^d^ Also adjusted for education category, social deprivation index, smoking status, alcohol consumption category, physical activity quintile, CVD, cancer or T2D at baseline, use of blood thinning medications, use of NSAID, TEI (KJ/day).
^e^ Also adjusted for BMI.
^f^ Also adjusted for multivitamin and multimineral use.
*Abbreviations: BMI, body mass index; NSAID, nonsteroidal anti-inflammatory drugs; CVD, cardiovascular disease; T2D, type 2 diabetes; TEI, total energy intake.*

**Supplementary Table S12 Hazard ratios and 95% confidence intervals between the top quartile of the Prime Diet Quality Score (PDQS) and mortality and morbidity outcomes among UK Biobank population subgroups^a^**

| **Subgroup** | **Mortality outcome** | | **Disease outcome** |
| --- | --- | --- | --- |
| Sex |  | Respiratory mortality |  |
| *Male* |  | 0.78 (0.51, 1.17)^b^ |  |
| *Female* |  | **0.48 (0.27, 0.85)** |  |
| *Corrected p-interaction^c^* |  | *.01* |  |
| Age group |  |  | CKD |
| <50 |  |  | 0.82 (0.57, 1.19) |
| 50-60 |  |  | **0.74 (0.62, 0.89)** |
| >60 |  |  | **0.74 (0.68, 0.81)** |
| *Corrected p-interaction^c^* |  |  | *<.0001* |
| Education level^d^ | Any mortality |  |  |
| *Low* | **0.82 (0.70, 0.95)** |  |  |
| *Other* | 0.85 (0.78, 0.94) |  |  |
| *Corrected p-interaction^c^* | *.005* |  |  |
| *Corrected p-interaction^c^* | *.001* | *.001* |  |
| Townsend deprivation index^e^ | Any mortality | Cancer mortality | CKD |
| *Low* | 0.93 (0.85, 1.03) | 0.95 (0.84, 1.08) | 0.78 (0.71, 0.87) |
| *High* | **0.74 (0.65, 0.83)** | **0.79 (0.68, 0.93)** | **0.68 (0.60, 0.77)** |
| *Corrected p-interaction^c^* | .001 | .03 | .004 |
| Smoker^f^ | Respiratory mortality | Cancer mortality |  |
| Never | 0.80 (0.47, 1.36) | 0.95 (0.82, 1.10) |  |
| Ever | **0.38 (0.24, 0.59)** | **0.78 (0.68, 0.90)** |  |
| *Corrected p-interaction^c^* | *.002* | *.03* |  |

^a^ Subgroup analyses were conducted only for statistically significant p-interaction values (corrected p<0.05).
^b^ All such values: HR (95%CI) of Q4 vs. Q1 PDQS quartile from fully adjusted models as using covariates equivalent to those used in the main models.
^c^ P-interaction obtained from an interaction term between the 1-SD PDQS and selected subgroups; p-values were corrected for false discovery rate using the Benjamini Hochberg procedure across mortality and morbidity category outcomes.
^d ”^Low” = CSEs or equivalent, O levels/GCSEs or equivalent; “other” = A levels and above.
^e^ Townsend deprivation index quintiles 1, 2 or 3 = “low”; quintiles 4 or 5 = “high”.
^f^ Current or past smoker = “ever smoker”.
*Abbreviations: CKD, chronic kidney disease; CSE, certificate of secondary education; CVD, cardiovascular disease; T2D, type 2 diabetes.*

**Supplementary Table S13 Sensitivity analysis showing hazard ratios and 95% confidence intervals between the
Prime Diet Quality Score (PDQS) and health outcomes in UK Biobank after removing the first two years of follow-up,
in fully adjusted models**

|  | **Q1** | **Q2** | **Q3** | **Q4** |
| --- | --- | --- | --- | --- |
| *All-cause mortality* |  |  |  | |
| HR (95% CI)^a^ | 1.00 (ref) | 0.95 (0.88, 1.02) | 0.84 (0.78, 0.90) | 0.85 (0.78, 0.91) |
| *T2D* |  |  |  |  |
| HR (95% CI) ^a^ | 1.00 (ref) | 0.90 (0.82, 0.99) | 0.78 (0.71, 0.85) | 0.73 (0.66, 0.81) |
| *CVD, any* |  |  |  |  |
| HR (95% CI) ^a^ | 1.00 (ref) | 0.91 (0.84, 0.98) | 0.87 (0.82, 0.93) | 0.88 (0.82, 0.95) |
| *Stroke, any* |  |  |  |  |
| HR (95% CI) ^a^ | 1.00 (ref) | 0.89 (0.77, 1.01) | 0.78 (0.68, 0.88) | 0.91 (0.80, 1.04) |
| *Respiratory disease, any chronic* | | | | |
| HR (95% CI) ^a^ | 1.00 (ref) | 0.94 (0.89, 0.99) | 0.89 (0.85, 0.93) | 0.87 (0.83, 0.92) |
| *NAFLD* |  |  |  |  |
| HR (95% CI) ^a^ | 1.00 (ref) | 0.94 (0.82, 1.09) | 0.78 (0.68, 0.90) | 0.66 (0.56, 0.78) |
| *CKD* |  |  |  |  |
| HR (95% CI) ^a^ | 1.00 (ref) | 0.88 (0.82, 0.95) | 0.79 (0.73, 0.84) | 0.74 (0.69, 0.80) |
| *Cancer, any* |  |  |  |  |
| HR (95% CI) ^a^ | 1.00 (ref) | 1.01 (0.95, 1.07) | 0.93 (0.88, 0.98) | 0.94 (0.89, 1.00) |
| *Lung cancer* |  |  |  |  |
| HR (95% CI) ^a^ | 1.00 (ref) | 0.95 (0.75, 1.21) | 0.77 (0.61, 0.97) | 0.71 (0.55, 0.92) |
| *Colorectal cancer* |  |  |  |  |
| HR (95% CI) ^a^ | 1.00 (ref) | 1.07 (0.90, 1.27) | 0.92 (0.78, 1.08) | 1.02 (0.85, 1.21) |
| *Prostate cancer* |  |  |  |  |
| HR (95% CI) ^a^ | 1.00 (ref) | 1.05 (0.94, 1.18) | 1.00 (0.90, 1.12) | 1.00 (0.89, 1.13) |
| *Postmen. breast can.* |  |  |  |  |
| HR (95% CI) ^a^ | 1.00 (ref) | 1.20 (1.02, 1.41) | 1.02 (0.87, 1.18) | 1.01 (0.87, 1.18) |
| *Neurodeg. disease, all* |  |  |  |  |
| HR (95% CI) ^a^ | 1.00 (ref) | 0.97 (0.86, 1.11) | 0.86 (0.76, 0.97) | 0.96 (0.85, 1.09) |
| *Parkinson’s disease* |  |  |  |  |
| HR (95% CI) ^a^ | 1.00 (ref) | 0.96 (0.77, 1.20) | 0.88 (0.71, 1.08) | 0.99 (0.79, 1.23) |
| *Dementia, all* |  |  |  |  |
| HR (95% CI) ^a^ | 1.00 (ref) | 1.00 (0.83, 1.21) | 0.92 (0.77, 1.10) | 1.08 (0.90, 1.29) |
| *Depression* |  |  |  |  |
| HR (95% CI) ^a^ | 1.00 (ref) | 0.88 (0.79, 0.97) | 0.86 (0.78, 0.95) | 0.94 (0.85, 1.04) |
| *Anxiety* |  |  |  |  |
| HR (95% CI) ^a^ | 1.00 (ref) | 0.89 (0.82, 0.97) | 0.85 (0.78, 0.92) | 0.85 (0.78, 0.92) |

^a^ Multivariable-adjusted model equivalent to model 3 for each outcome.

*Abbreviations: CVD, cardiovascular disease; T2D, type 2 diabetes; CKD, chronic kidney disease; NAFLD, non-alcoholic fatty liver disease.*

**
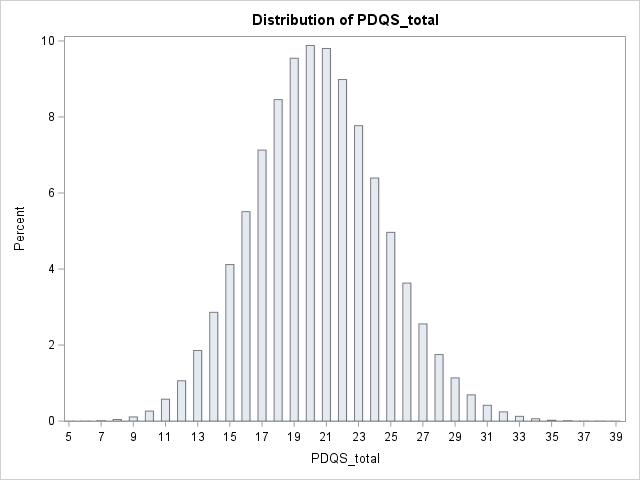
Supplementary Fig. S2 Distribution of the total Prime Diet Quality Score (PDQS)**

**Supplementary Fig. S3 Cubic spline graph of fully adjusted models between the Prime Diet Quality Score (PDQS) and health outcomes**

| 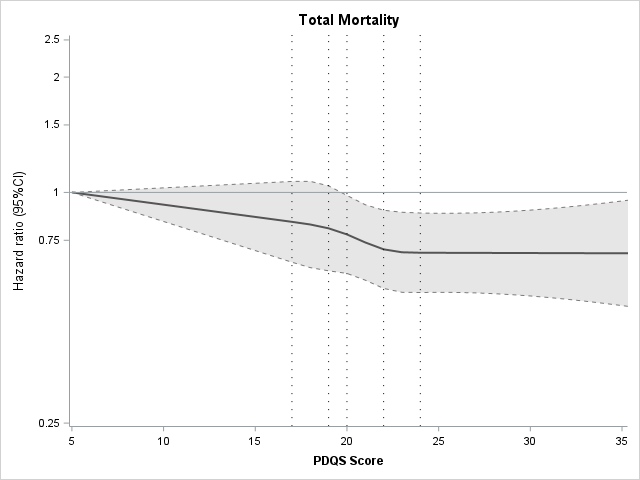 | 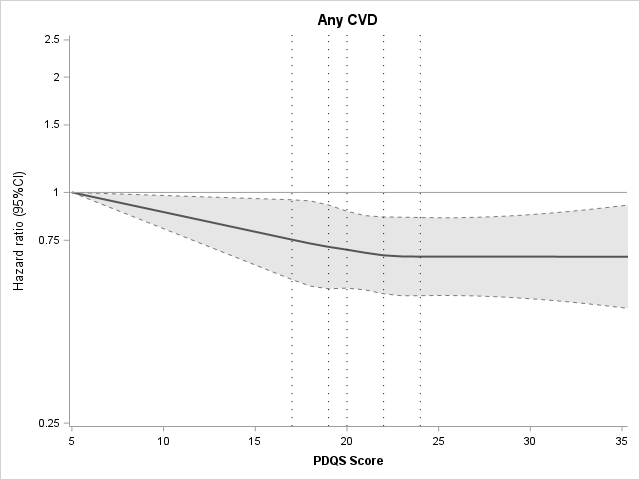 |
| --- | --- |
| p=0.83 | p=0.88 |
| 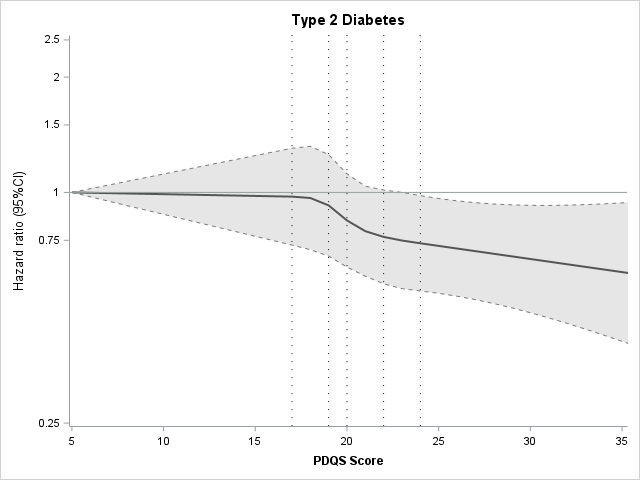 | 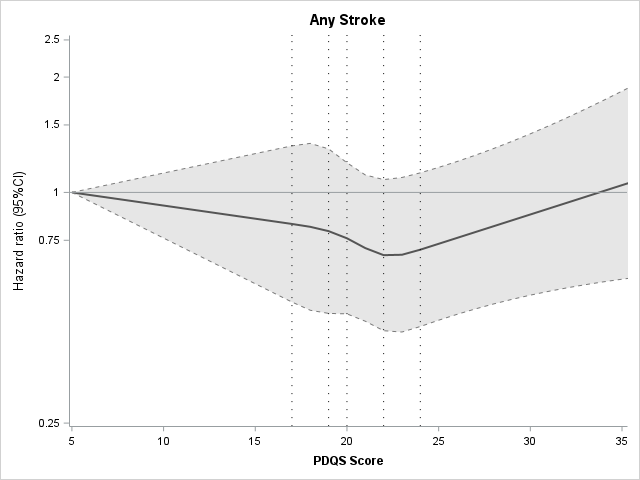 |
| p=0.36 | p=0.84 |
| 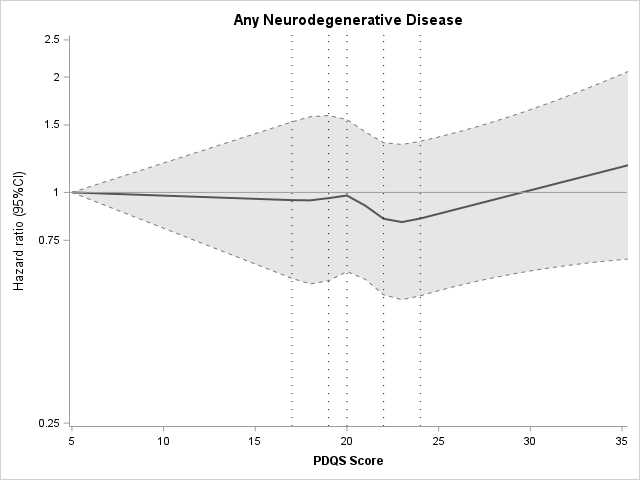 | 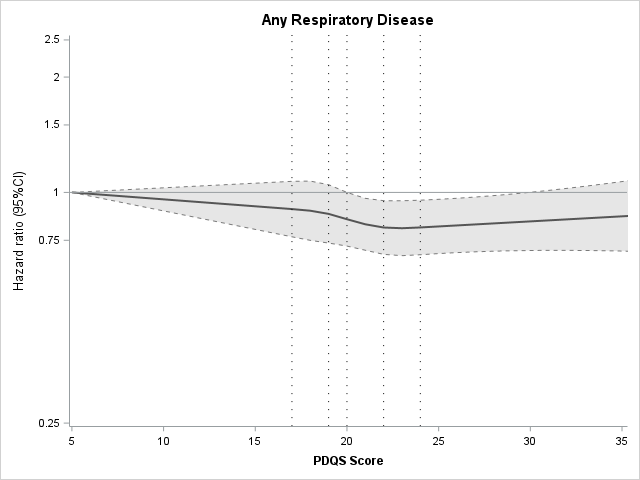 |
| p=0.24 | p=0.86 |
| 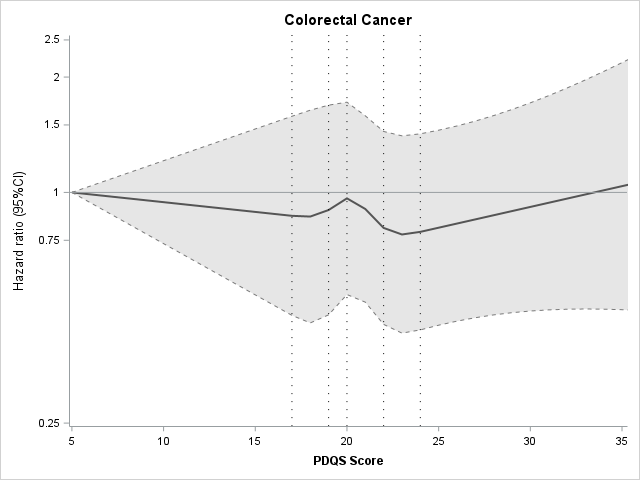 | 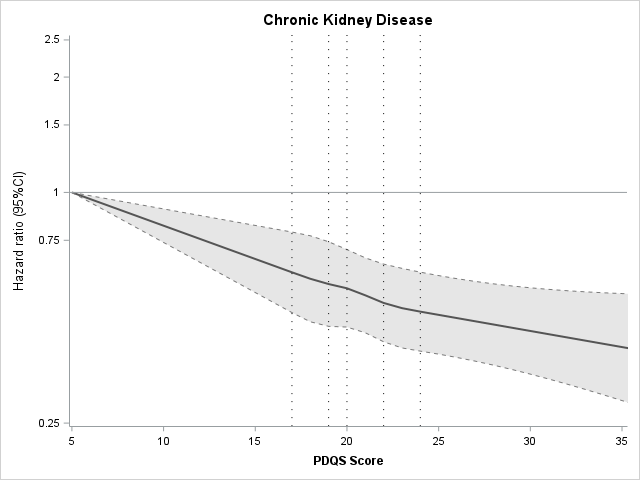 |
| p=0.14 | p=0.62 |
| 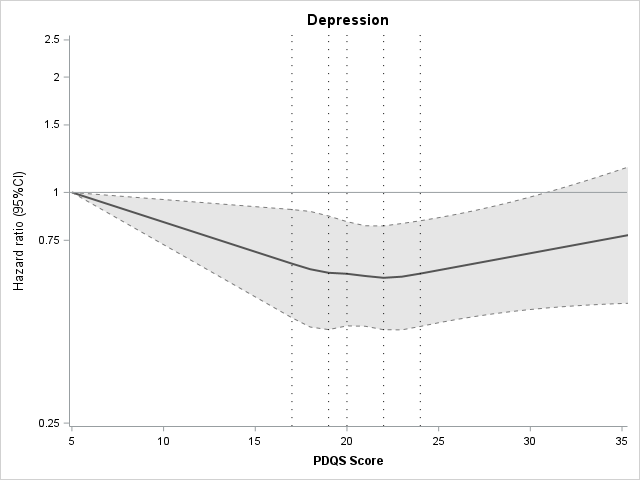 | 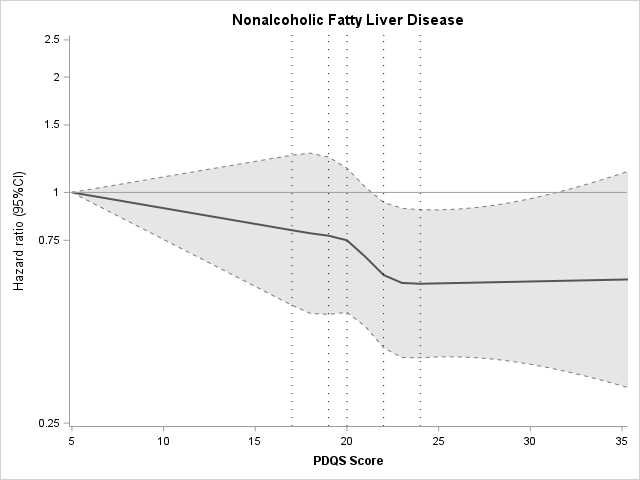 |
| p=0.71 | p=0.42 |

Multivariable Cox regression models with restricted cubic splines for associations between the PDQS and health outcomes, knots placed at quintile medians.

**Supplementary references**

1. Brennan, S.F., et al., *Validity and reproducibility of the Prime Diet Quality Score (PDQS) against a four-day food diary in adults at risk of cardiovascular disease on the island of Ireland.* Proceedings of the Nutrition Society, 2022. **81**(Oce4) DOI: Artn E97

Pii S0029665122001264

10.1017/S0029665122001264: p.

2. Kronsteiner-Gicevic, S., et al., *Validation of the Rapid Prime Diet Quality Score Screener (rPDQS), A Brief Dietary Assessment Tool With Simple Traffic Light Scoring.* J Acad Nutr Diet, 2023. **123**(11) DOI: 10.1016/j.jand.2023.05.023: p. 1541-1554 e7.

3. Gicevic, S., et al., *Diet quality and all-cause mortality among US adults, estimated from National Health and Nutrition Examination Survey (NHANES), 2003-2008.* Public Health Nutr, 2021. **24**(10) DOI: 10.1017/S1368980021000859: p. 2777-2787.

4. Thompson, D.J., et al., *UK Biobank release and systematic evaluation of optimised polygenic risk scores for 53 diseases and quantitative traits.* medRxiv, 2022 DOI: 10.1101/2022.06.16.22276246: p. 2022.06.16.22276246.
